# Synthesis and Characterization of Conjugated Hyaluronic Acids. Application to Stability Studies of Chitosan-Hyaluronic Acid Nanogels Based on Fluorescence Resonance Energy Transfer

**DOI:** 10.3390/gels8030182

**Published:** 2022-03-15

**Authors:** Volodymyr Malytskyi, Juliette Moreau, Maité Callewaert, Céline Henoumont, Cyril Cadiou, Cécile Feuillie, Sophie Laurent, Michael Molinari, Françoise Chuburu

**Affiliations:** 1 Institut de Chimie Moléculaire de Reims, University of Reims Champagne Ardenne, CNRS, ICMR UMR 7312, 51097 Reims, France; juliette.moreau@univ-reims.fr (J.M.); maite.callewaert@univ-reims.fr (M.C.); cyril.cadiou@univ-reims.fr (C.C.); 2 Institut Parisien de Chimie Moléculaire, Sorbonne Université, CNRS, IPCM UMR 8232, 4 Place Jussieu, 75252 Paris, France; 3 NMR and Molecular Imaging Laboratory, University of Mons UMons, B-7000 Mons, Belgium; celine.henoumont@umons.ac.be (C.H.); sophie.laurent@umons.ac.be (S.L.); 4 Center for Microscopy and Molecular Imaging, Rue Adrienne Bolland 8, B-6041 Charleroi, Belgium; cecile.feuillie@u-bordeaux.fr (C.F.); michael.molinari@u-bordeaux.fr (M.M.); 5 Institut de Chimie et Biologie des Membranes et des Nano-Objets, CNRS UMR 5248, University of Bordeaux, IPB, 33600 Pessac, France

**Keywords:** nanohydrogels–hyaluronic acid–HA-mPEG_2000_, fluorinated and fluorescent HA conjugates–hyaluronic acid degree of substitution–diffusion ordered spectroscopy (DOSY)–1D diffusion-filtered ^19^F NMR–atomic force microscopy–FRET experiments–hyaluronidase–nanohydrogel stability

## Abstract

Hyaluronic acid (HA) was functionalized with a series of amino synthons (octylamine, polyethylene glycol amine, trifluoropropyl amine, rhodamine). Sodium hyaluronate (HAs) was first converted into its protonated form (HAp) and the reaction was conducted in DMSO by varying the initial ratio (−NH_2_ (synthon)/COOH (HAp)). HA derivatives were characterized by a combination of techniques (FTIR, ^1^H NMR, 1D diffusion-filtered ^19^F NMR, DOSY experiments), and degrees of substitution (DS_HA_) varying from 0.3% to 47% were determined, according to the grafted synthon. Nanohydrogels were then obtained by ionic gelation between functionalized hyaluronic acids and chitosan (CS) and tripolyphosphate (TPP) as a cross-linker. Nanohydrogels for which HA and CS were respectively labeled by rhodamine and fluorescein which are a fluorescent donor-acceptor pair were subjected to FRET experiments to evaluate the stability of these nano-assemblies.

## 1. Introduction

Since its first isolation in 1934 from the vitreous humor of bovine eyes, hyaluronic acid (**HA**) has been used in many applications and research areas [1]. This unbranched glycosaminoglycan which is composed of repeating units of disaccharides N-acetyl glucosamine (GlcNAc) and D-glucuronic acid (GlcA) linked together through alternating β-1,3 and β-1,4 glycosidic bonds, is a negatively charged polymer [2] at physiological pH (3 < pKa (COOH groups on the D-glucuronic acid residues) < 4). **HA** molecules strongly bind to water molecules and become heavily hydrated to form a viscous gel. This property is at the origin of the viscoelastic character and the control of tissue hydration [3,4] and, as a primary component of extracellular matrix (ECM) vitreous humor and synovial fluid of vertebrates, functions as a scaffold for the organization of these biofluids. **HA** has become a carrier of great interest not only owing to its advantages such as biodegradability, biocompatibility, but also to its intrinsic targeting properties, based on the selective interactions with receptors, such as CD44 or hyaluronan receptors for endocytosis (HARE) [5]. For these reasons, exogenous **HA** has been investigated as a drug delivery system for therapeutics and diagnostics [3,4,5,6,7,8,9,10,11,12,13,14]. 

To improve its properties and target its applications, **HA** can be subjected to chemical modifications. To do this, synthetic approaches are mainly based on (a) the functionalization of a carboxylic acid group by peptide coupling [15,16], esterification [17], or Ugi condensation reaction [18] (b) the functionalization of hydroxyl groups by alkylation [19] or acylation [20,21] or (c) a partial oxidative degradation of the polymer [22]. It is worth mentioning that between these methods, peptidic coupling is the most commonly used due to the accessibility of amine functions for the introduction of various side-groups and due to the robust nature of the amide bond formed. The commercially available bio-extracted sodium hyaluronate (**HAs**) is a water-soluble polyanionic polymer. Its chemical modification by peptidic coupling reaction is typically carried out using conventional coupling agents such as EDC/NHS in an aqueous medium [23,24]. However, these latter form in situ activated intermediates that can be hydrolyzed by water molecules prior to their reaction with amines [25,26,27]. This usually determines low yields of grafting in water and requires the use of a significant excess of coupling agents and amines. Another drawback of this strategy is the difficulty to evidence the formation of the amide bond because the amide protons are often invisible when NMR is performed in protic solvents. An alternative is to carry out the reaction in organic aprotic solvents and under anhydrous conditions. Palumbo et al. [28] recently showed the possibility to manipulate hyaluronic acid in a pure organic solvent, such as DMSO, by the transformation of **HAs** into its tetrabutylammonium (TBA^+^) salt and its further activation using 4-NPBC which is completely unstable in water. However, the application of functionalized polysaccharides in the nanomedicine field subsequently requires their solubility in water, and therefore an additional step to make cation exchange again (TBA^+^ to Na^+^) is necessary. The alternative is to use a protonated form of hyaluronic acid (**HAp**) because this polymer is simultaneously soluble both in DMSO and in water unlike the **HAs** form, and the peptide coupling in DMSO allows the straightforward determination of the degree of substitution by NMR. To our knowledge, only two examples of peptidic coupling using such an approach can be found in the literature to date [29,30]. 

In this context and in order to extend the scope of this method we have systematically reinvestigated **HAp** functionalization, in which the level of **HAp** substitution was varied (by increasing the initial synthon/COOH HA molar ratio), characterized by FTIR and quantified by a combination of NMR techniques. The method was developed from the model functionalization reaction between **HAp** and *n*-octylamine, using HATU (1-[bis(dimethylamino)methylene]-1H-1,2,3-triazolo [4,5-b]pyridinium 3-oxid hexafluoro-phosphate) as a coupling agent, a method which allows to graft different ligands of interest for **HA** such as fluorinated synthons and PEG moieties (stealthiness) and fluorescent tags (rhodamine for fluorescence imaging). Moreover, we have demonstrated that nanohydrogels (NGs) obtained by ionic gelation between **HA** and chitosan (**CS**) in the presence of tripolyphosphate (TPP) as a cross-linking agent are particularly well suited to encapsulate gadolinium chelates (**GdCAs**) and tremendously increase the efficiency of these paramagnetic MRI probes [31,32,33,34]. Ionic gelation relies on the development of electrostatic interactions between the negative charges of hyaluronic acid and the positive charges of chitosan. Therefore, special attention must be paid to the HA degree of substitution (DS_HA_) in order to ensure that after functionalization, there are enough negative charges left on the functionalized HA for the establishment of these interactions (which must remain sufficient for the ionic gelation to still lead to the formation of functionalized nanoparticles). Furthermore, when it comes to nanogels obtained by ionic gelation, the question of their stability is raised. In order to answer this question, we will use the functionalization of **HAs** developed herein with rhodamine (**HA-Rhod**) to elaborate nanogels with recently fluorescein-labeled chitosan (**CS-Fluo**) and (i) evaluate by fluorescence spectroscopy the occurrence of a Förster resonance transfer (FRET) signal within nanogels and (ii) test the conditions of degradation of the edifice, in particular in the presence of enzymes [35,36].

## 2. Results and Discussion

### 2.1. Chemical Functionalization of Hyaluronic Acid

**Scheme 1:**
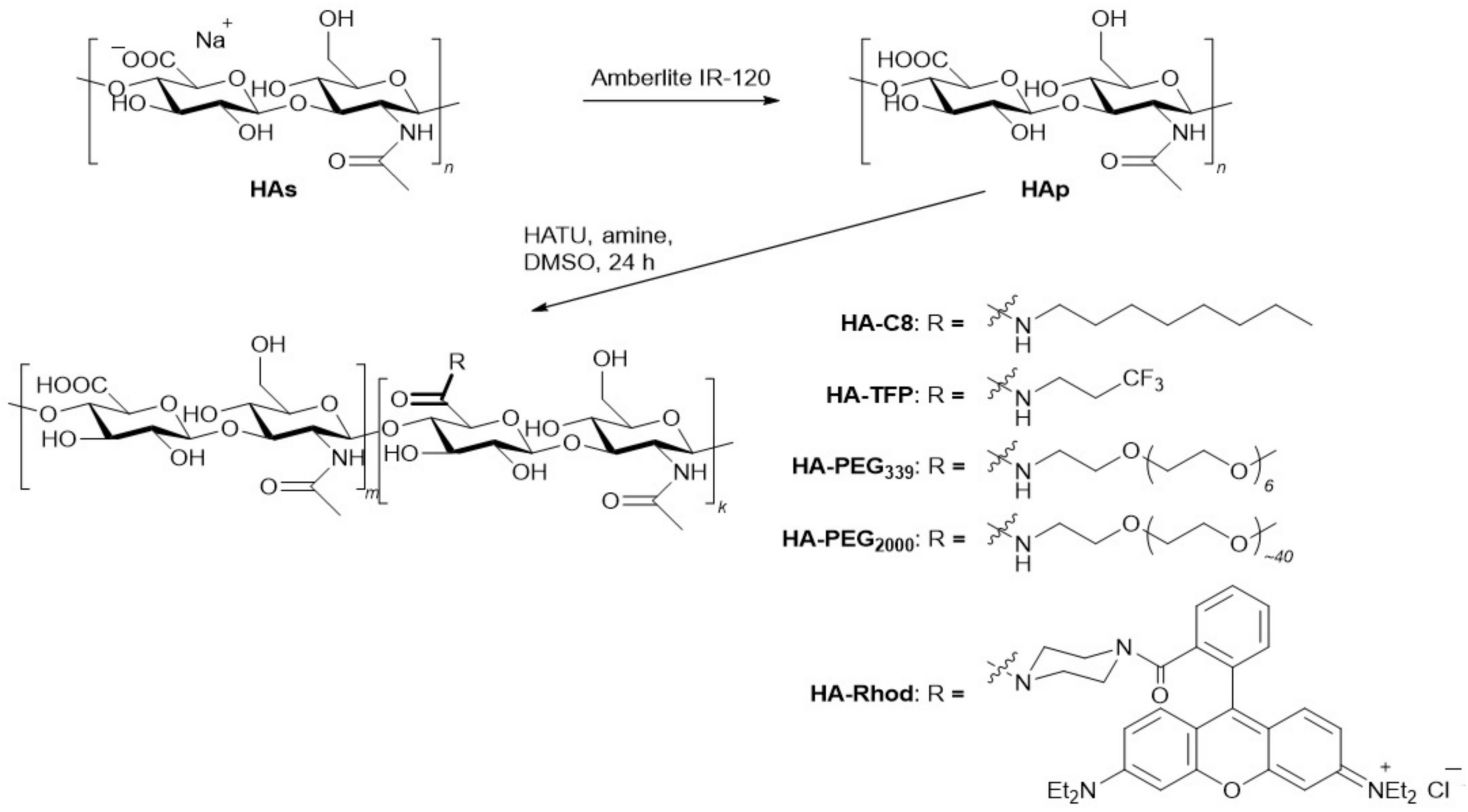
Syntheses of functionalized HA described in the paper.

The functionalization of HA by peptidic coupling was run in DMSO under anhydrous conditions in order to avoid any hydrolysis of the intermediates which could be detrimental to the performance of functionalization [25,26,27]. This implies beforehand to improve the solubility of HA in this solvent. For that, the commercially available sodium hyaluronate HAs was converted into its protonated form **HAp** by column exchange chromatography, according to the procedure of Vasi et al. [20]. After complete dehydration by lyophilization, **HAp** was used in a peptidic coupling reaction in anhydrous DMSO using **HATU** as a coupling agent. A series of amines have been used in the general synthetic method as illustrated in Scheme 1.

Five amino synthons were used to functionalize **HAp**, *n*-octylamine **C8-NH_2_** (as a model to set up the synthesis and characterization protocols), 1,1,1-trifluoropropylamine **TFP-NH_2_**, 2,5,8,11,14,17,20-heptaoxadocosan-22-amine **PEG_339_-NH_2_**, methoxy-poly(ethylene)glycol-amine **PEG_2000_-NH_2_**, and amine-functionalized rhodamine **Rhod-NH.** The molar ratios (−NH_2 synthons_ to −COOH _HA_) were initially fixed at 10% (condition **a**), 20% (condition **b**), 50% (condition **c**), and 100% (condition **d**), respectively, for each amino synthon. The reactions were carried out under ambient conditions for one day. Functionalized polymers were precipitated from the organic solution, purified by ultrafiltration to eliminate all the unreacted low-molecular-weight compounds, and freeze-dried prior to their characterization by FTIR and NMR methods.

#### 2.1.1. Grafting of *n*-Octylamine on Hyaluronic Acid

**Figure 1:**
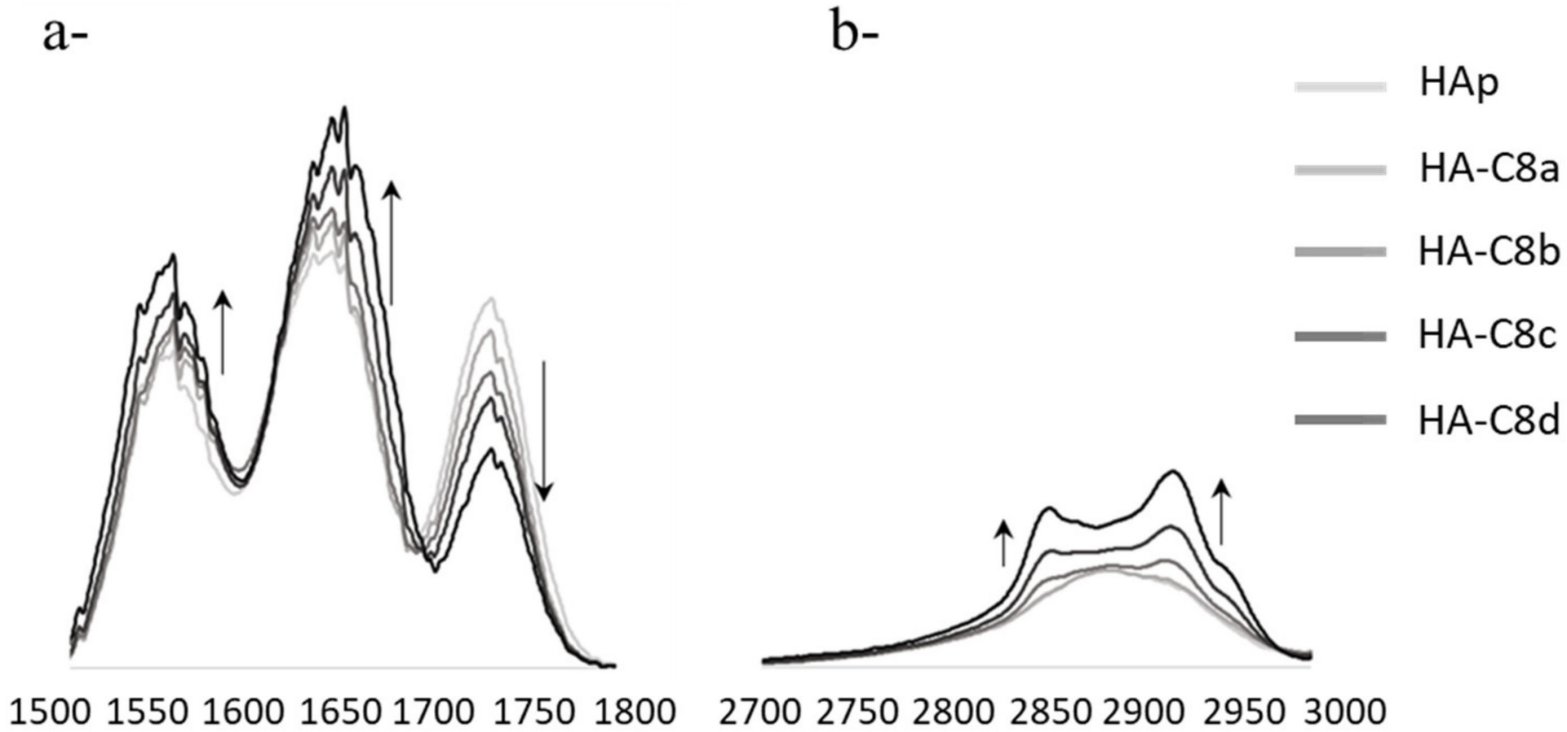
FTIR spectra of octylamine functionalized HA samples: (**a**) carbonyl vibrations region (1500–1800 cm^−1^) and (**b**) C–H stretching vibrations region (2700–3000 cm^−1^).

*n*-Octylamine (**C8-NH_2_**) is a simple and accessible product that allows the introduction of an alkyl chain into a polysaccharide backbone. This latter can be easily identified by ^1^H NMR and FTIR analysis and as such, can be used as a model to develop the conditions for **HA** functionalization and characterization of functionalized **HA**. At the same time and from an applicative point of view, hydrogels obtained with HA derivatized with such alkyl chains are known to exhibit improved viscoelastic properties and increased resistance to enzymatic hydrolysis [28]. After functionalization, Haps functionalized by octylamine (**HA-C8** polymers) revealed a modification of their solubility: **HA-C8a**, **HA-C8b**, and **Ha-C8c** were water soluble, **HA-C8c** was also soluble in chloroform, while **HA-C8d** was insoluble in water. After purification by ultrafiltration (**HA-C8a**, **HA-C8b**, and **HA-C8c**) or centrifugation (**HA-C8d**), all the samples were characterized by FTIR spectroscopy (Figure 1). A clear change in HA carbonyl vibration patterns was observed. As the amount of amine increased, a clear growth in the amide I band (ν(C=O), centered at 1644 cm^−1^) was observed. It was accompanied by the same enhancement of the amide II band (ν(C–N), centered at 1557 cm^−1^), confirming the increase in the quantity of amide groups in samples **HA-C8a-d** and therefore, a rise in the degree of functionalization. At the same time, the intensity of the HA carboxylic acid band (ν(C=O), centered at 1732 cm^−1^) consistently decreased. The increasing quantity of grafted alkyl chains was also confirmed by the C–H vibration band enhancement. Two distinct peaks were rising at 2857 cm^−1^ and 2925 cm^−1^, corresponding, respectively, to the symmetrical and the asymmetrical stretching vibrations of −CH_2_ groups. The last band also exhibited a shoulder around 2953 cm^−1^ which could be attributed to the asymmetrical C–H stretching vibrations of the −CH_3_ group.

**Figure 2:**
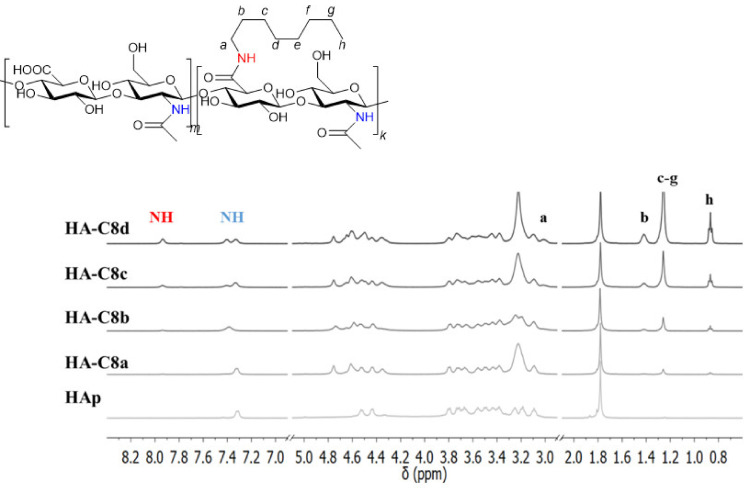
^1^H NMR spectra of octylamine functionalized HA: **Hap**, **HA-C8a**, **HA-C8b**, **HA-C8c**, and **HA-C8d**) (500 MHz, 318 K, DMSO *d*_6_, and DMSO peak is omitted for clarity).

In order to evaluate the grafting degree and then, the degree of substitution of HA by octylamine, **HA-C8** polymers were subjected to a ^1^H NMR analysis (318 K, DMSO *d*_6_, Figure 2).

In these spectra (**HA-C8a** to **HA-C8d**), the rise of the alkyl chain protons at 0.87 ppm (C*H*_3_), 1.26, 1.42, and 3.02 ppm (C*H*_2_) was clearly observed, with the increasing quantity of amine. However, contrary to the commonly accepted opinion, they did not necessarily correspond to grafted synthons but can also come from octylamine (or octylammonium) associated via electrostatic interactions to a polyanionic polymer such as **HAp** [37]. The integration of one of these signals in comparison to one of the HA signals, however, led to the determination of the association rate (AR) of the C8 chain to HA (Table 1, entry 1). The most straightforward method to quantify the degree of substitution of HA (DS_HA_), and then the grafting efficiency, consisted in integrating amide proton peaks (Table 1, entry 2). Indeed, while more and more carboxyl groups were modified, the splitting of the acetamide signal at 7.4 ppm was observed as well as the occurrence of a new signal at 7.9 ppm, that corresponded to the newly formed amide. Such an observation was only possible using DMSO-*d*_6_ as a solvent and not in a solvent for which NH proton signals cannot be detected due to their exchangeable nature.

These results were corroborated by DOSY experiments (see Supplementary Materials, Figure S1). As described in the experimental part, the fitting of the diffusion curves of **HA-C8** extracted from DOSY experiments allowed to determine the fraction of C8 covalently grafted to HA. For **HA-C8a** and **HA-C8b**, a biexponential curve was obtained, and its fitting with Equation (1) allowed to extract the percentage of grafted C8 over the total amount of C8 (71% and 92% for **HA-C8a** and **HA-C8b**, respectively). For HA-C8c and HA-C8d, a monoexponential curve was obtained, and its fitting gave a diffusion coefficient equal to that of HA, proving that 100% of C8 was covalently grafted to HA.

The DS values obtained for **HA-C8** by both methods (by integrating NH peaks or by integrating aliphatic peaks corrected with DOSY analysis) were found to be close (±2–4%) and linearly dependent on the amount of amine initially introduced in the reaction medium (see Supplementary Materials, Figure S2).

#### 2.1.2. Grafting of Polyethyleneglycol Oligomers on Hyaluronic Acid

Polyethyleneglycol (PEG) synthons are often used to improve nanostructure stealthiness in biological media [38,39,40,41]. In this context, the grafting of two PEG amines was tested, one bearing exclusively seven ethylene glycol residues of (**PEG_339_-NH_2_**) and the other being a mixture of larger oligomers with an average molar mass of 1834 Da (**PEG_2000_-NH_2_**), i.e., approximately 40 ethylene-oxy residues (Scheme 1). HA functionalization was subsequently followed by FTIR and ^1^H NMR spectroscopies, according to increasing initial quantities of PEG amines (**HA-PEG_339_a-d** and **HA-PEG_2000_a-d** polymers, respectively). The FTIR spectra of **HA-PEG_339_a-d** and **HA-PEG_2000_a-d** confirmed the successful modification of HA with oligomer PEG chains (see Supplementary Materials, Figures S3 and S4, and related commentaries).

As previously demonstrated for **HA-C8** polymers, ^1^H NMR spectroscopy in DMSO-*d*_6_ allowed the determination of the DS_HA_ with PEG moieties. The signals corresponding to oligomeric ethylene glycol units and terminal methoxy groups were clearly observed at 3.51 ppm and 3.24 ppm respectively (see Supplementary Materials, Figures S5 and S6) but, due to an overlap with peaks of **HA** backbone, these signals cannot be used to quantify the extent of functionalization. In these conditions, DS_HA_ were determined by the integration of amide proton signals at 8.10 ppm for each copolymer (Table 2).

The DS_HA_ obtained for **HA-PEG** polymers were in the same order of magnitude as the ones determined for **HA-C8** polymers It is interesting to notice that (i) the variation of DS_HA_ according to initial amounts of pegylated amine introduced in the preparation was again linear (see Figures S7 and S8) and (ii) the DS_HA_ measured with PEG synthons were in the same order of magnitude as the ones determined with the lipophilic C8 chain.

#### 2.1.3. Grafting of Trifluoropropylamine on Hyaluronic Acid

The introduction of fluorinated groups is considered as a solution in pharmaceutical chemistry for improving the lipophilicity of active substances and their subsequent accumulation in lymph nodes [37,42]. For this reason, the synthetic method developed herein was extended to the introduction of fluorinated groups on HA by means of the commercially available 1,1,1-trifluoropropylamine (**TFP-NH_2_**, Scheme 1). After synthesis and purification, the successful grafting was evidenced by FTIR (see, Figure S9) and ^1^H NMR (see Supplementary Materials, Figure S10).

**Figure 3:**
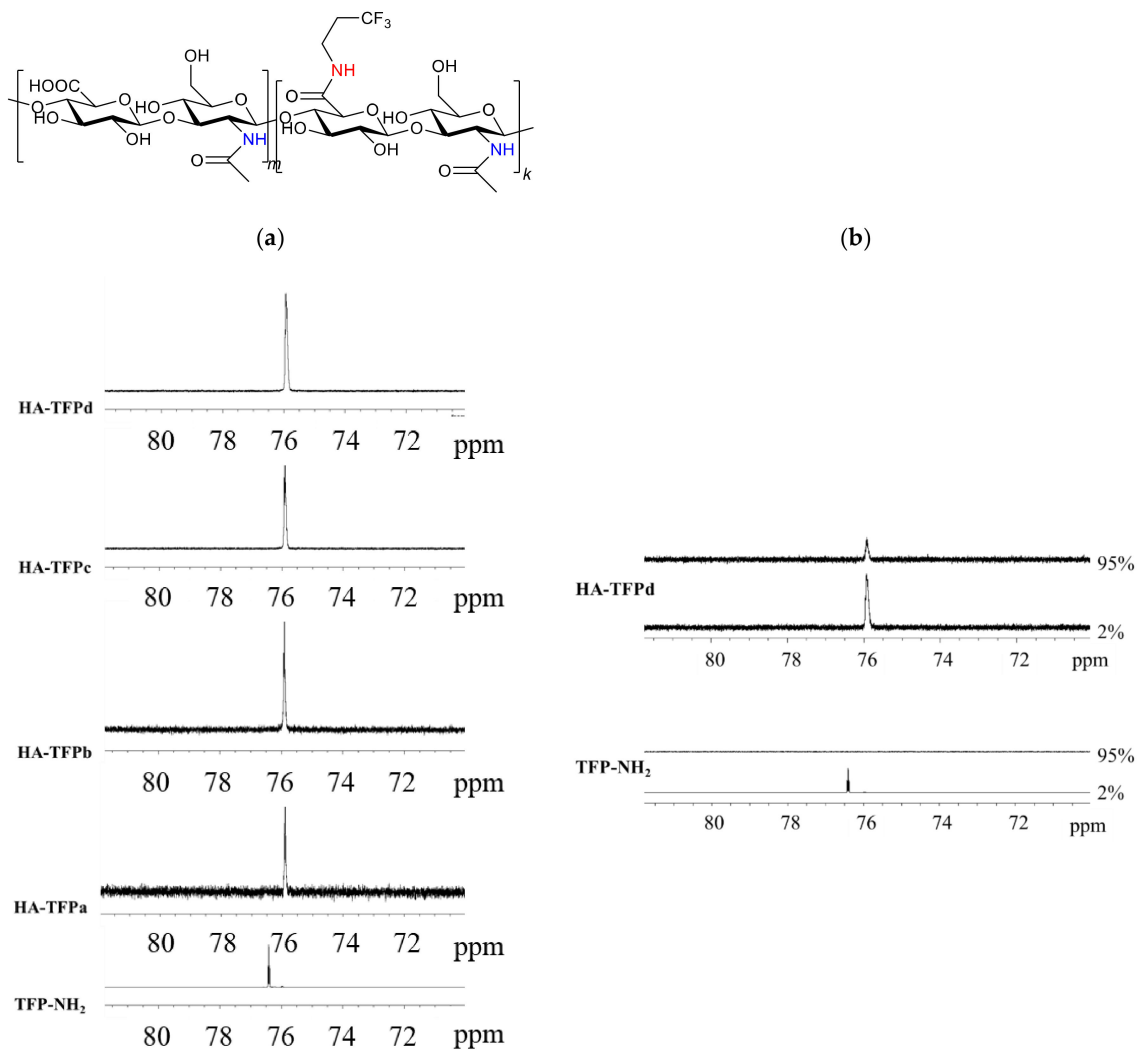
(**a**) ^19^F NMR spectra of trifluoropropyl functionalized HA: **HA-TFPa**, **HA-TFPb**, **HA-TFPc** and **HA-TFPd** for initial molar ratios (NH_2_/COOH) = 10 (bottom), 20, 50, and 100 (top) %, respectively; **TFP-NH_2_** ^19^F spectrum is given as a reference (470.64 MHz, 318 K, DMSO-*d_6_*). (**b**) One-dimensional (1D) diffusion-filtered ^19^F NMR spectra of **HA-TFPd** and **TFP-NH_2_** with a gradient g of 2% and 95%.

Thanks to the presence of terminal CF_3_ groups, evidence of grafting was also obtained in ^19^F NMR spectroscopy (Figure 3).

As soon as the **TFP-NH_2_** compound is grafted onto HA, a strong-field shift of CF_3_ signal occurs, from 76.4 ppm (ungrafted **TFP-NH_2_**) to 75.9 ppm (**HA-TFP**) (Figure 3a). One-dimensional (1D) diffusion-filtered ^19^F spectra concomitantly recorded (Figure 3b and see also Supplementary Materials Figure S11) showed that for all the **HA-TFP** samples, the application of a 95% diffusion filter was accompanied by the permanence of the ^19^F signal, while for **TFP-NH_2_** the application of the same filter induced the disappearance of the signal. Under these applied filtering conditions, species that quickly diffuse are removed, while the 2% gradient condition is not able to discriminate between low and rapid diffusion species. Consequently, for all **HA-TFPa-d** samples, the peak at 75.9 ppm was unambiguously assigned to the signal of TFP grafted to the HA backbone.

The quantification of DS_HA_ in **HA-TFPa-d** samples was then performed by ^1^H NMR spectroscopy. As previously noticed for HA-PEG copolymers, the ^1^H signals of the newly grafted −(CH_2_)_2_ chain were masked by the peaks of the polymer. Therefore, the quantification of DS_HA_ was again performed by integrating the ^1^H signal of the amidic proton associated with the newly formed peptide bond at 8.25 ppm (Table 3). Compared to C8- and PEG-functionalized HA, a slight low-field shift of the amidic proton signal was observed due to the electron-withdrawing effect of the trifluoromethyl group. Obtained DS values were gathered in Table 3 (see also Supplementary Materials, Figure S12).

#### 2.1.4. Grafting of Rhodamine B Amine on Hyaluronic Acid

An important property for nanomaterials designed for the biomedical field is their ability to be tracked in vivo, particularly by fluorescence imaging. In the current study, this requires the control of the HA grafting reaction by fluorescent synthons. We have chosen as a model fluorophore the rhodamine **Rhod-NH** [43] which was introduced on the HA skeleton by peptidic coupling. (Scheme 1) Therefore, HA backbone functionalization was performed with rhodamine and the corresponding conjugates characterized as above to obtain a precise evaluation of the grafting rate and then of DS_HA_.

The efficiency of the grafting was firstly followed by an FTIR analysis (see Supplementary Materials, Figure S13).

Unlike the four synthons described above, **Rhod-NH** is a secondary amine. Therefore, after its grafting to HA, the amide formed is tertiary and bears no proton, which makes the characterization more challenging. As a result, the ^1^H NMR spectra of **HA-Rhod** polymers (see Supplementary Materials, Figure S14) showed that there was no newly arising amide proton signal at low fields, and the only clearly distinctive synthon-related peaks were related to the aromatic protons (between 6.4 and 8.1 ppm) and to the methyl protons (at 1.2 ppm).

**Figure 4:**
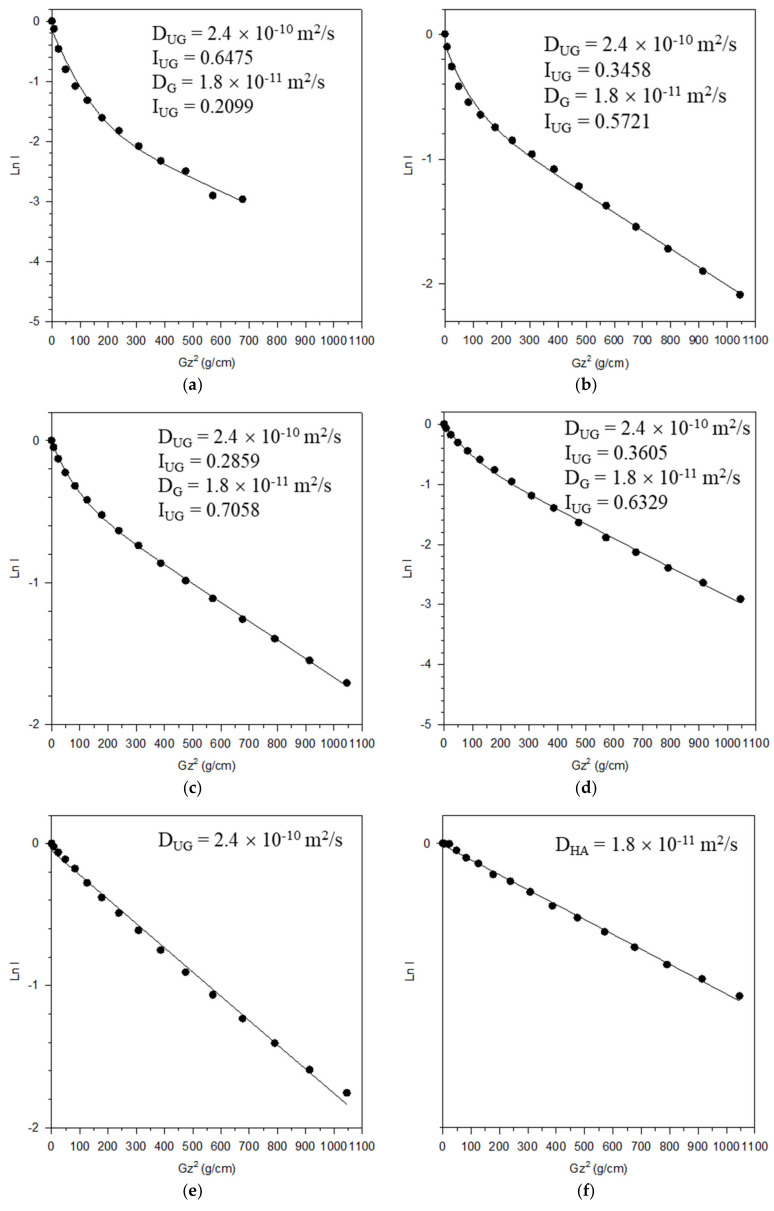
Diffusion curves and diffusion coefficients extracted from DOSY spectra (ref δ (^1^HRhod) = 1.1 ppm) for (**a**)—**HA-Rhod a**, (**b**)—**HA-Rhod b**, (**c**)—**HA-Rhod c**, and (**d**)—**HA-Rhod d** and (**e**) Rhod-NH alone and (**f**) HA alone as controls. See Equation (1) for I_UG_ and I_G_ definitions (UG corresponds to ungrafted Rhod synthon, while G corresponds to grafted Rhod synthon).

Although the latter signal at the high field was quite intense (corresponding to 12 H from two diethylamino groups of rhodamine) and well-suitable to quantification (well-separated from the other signals), it did not necessarily correspond to the grafted rhodamine only, as already discussed for **HA-Rhod** polymers. Indeed, at this level, it was not possible to distinguish between associated (by electrostatic interactions) and grafted Rhod synthons. That is why **HA-Rhod** polymers were subjected to DOSY experiments and were carried out to determine the grafting degree of rhodamine synthons on HA chains (Figure 4).

The obtained diffusion curves were clearly nonlinear (Figure 4). A biexponential fitting of these curves with Equation (1) was performed. The first coefficient of 2.40 × 10^−10^ m^2^ s^−1^ corresponded to ungrafted Rhod synthons that quickly diffused, and the second one (1.8 × 10^−11^ m^2^ s^−1^), corresponding to Rhod synthons that diffused much more slowly, was attributed to grafted functionalized rhodamine moieties. For the latter, the diffusion coefficient was the same as that of HA (see experimental section). This was expected because rhodamine and HA chains have very different molecular weights, and rhodamine grafting should not restrict HA chain mobility. In a second step, this fitting allowed the extraction of the percentage of grafted rhodamine over the total amount of rhodamine (grafting degree (GD) Rhod_G_/Rhod_T_, Equation (2), experimental section, and Table 4). Finally, the combination of ^1^H NMR integration (of the peak at 1.2 ppm) and DOSY analysis allowed to obtain the final DS_HA_ (Table 4).

The variation of DS_HA_ according to initial amounts of rhodamine introduced in the preparation was also linear here (see Supplementary Materials, Figure S15).

### 2.2. Nanogel Syntheses with Functionalized HA and Characterization—Evaluation of Nanogel Stability by Förster Energy Transfer Experiments (FRET)

#### 2.2.1. Nanogel Synthesis with Functionalized HA and Characterization

Functionalized HA polymers in association with chitosan (CS) were used to produce nanoparticles by physical gelation, in a one-step procedure. This method relied upon the establishment of multivalent electrostatic interactions between HA derivatives (polyanionic) and CS (polycationic). The resulting supramolecular network could be reinforced by cross-linking mediated by small anionic cross-linkers such as sodium tripolyphosphate (TPP) [44]. Functionalized HA with various DS_HA_ were then evaluated for their ability to produce functionalized CS-TPP/HA NPs by ionic gelation. Functionalized CS-TPP/HA nanogels formation was evidenced by DLS. The average hydrodynamic diameters of NPs were determined by dynamic light scattering (DLS, Table 5) recording hydrodynamic diameters and polydispersity index (PDI) of the nanosuspensions. Nanoparticle zeta potential (ζ) which was indicative of their outermost surface charge was determined by ELS.

DLS experiments showed the presence of relatively monodisperse nanoassemblies (PDI ≤ 0.35) whose size varied from 130 to 155 nm. For some samples, AFM images in liquid mode corroborated the formation of nanoparticles by evidencing nanoassemblies of lower size (30–70 nm) and the presence of some aggregates (see Supplementary Materials, Figure S16). Such differences between DLS and AFM measurements have already been observed for nanogels [45] and attributed to the fact that in DLS, because of the presence of aggregates, the response could be biased by the use of mathematical models of signal processing. For **CS-TPP/HA-Rhod** nanogels, the confocal image and the associated fluorescence spectrum exhibited the expected features for the **CS-TPP/HA-Rhod** NGs, confirming the fact that the NGs are fluorescent (see Supplementary Materials, Figure S17).

#### 2.2.2. Evaluation of Nanogels Stability by FRET Experiments

**Figure 5:**
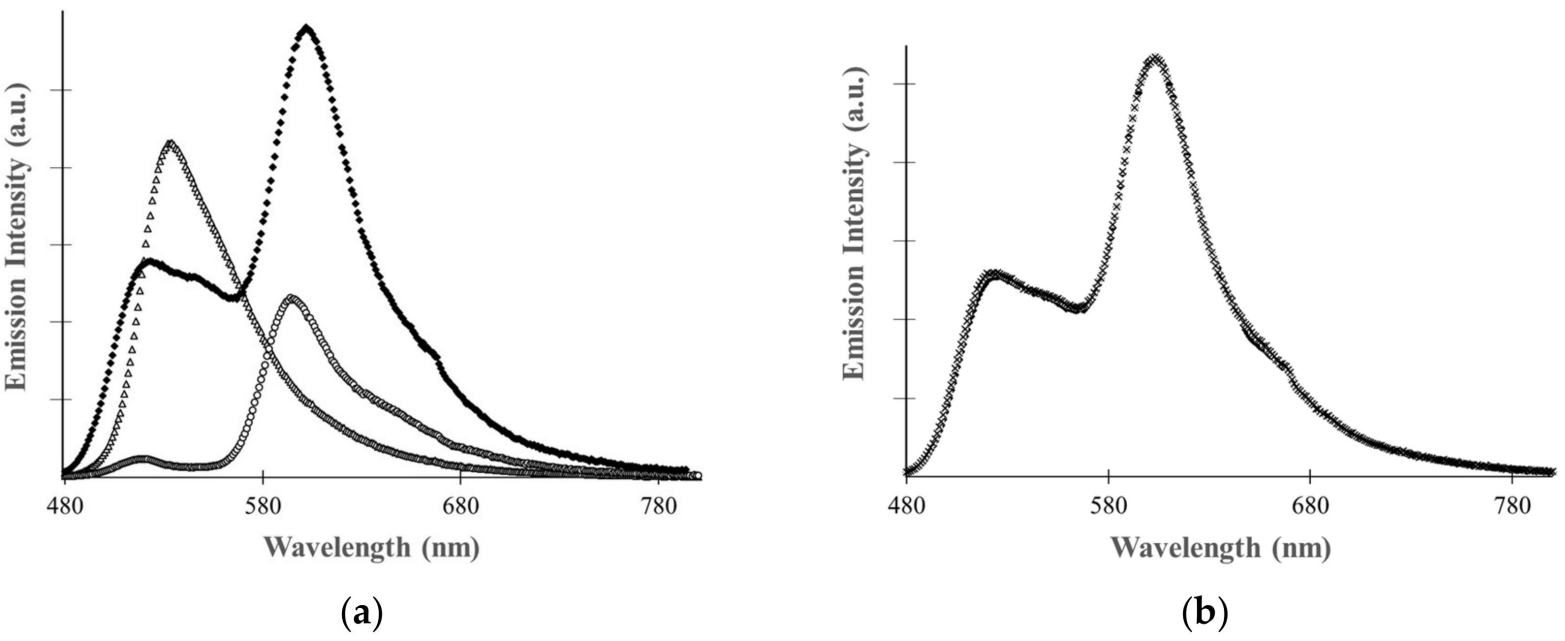
(**a**) Emission spectrum of **CS-Fluo-TPP/HA-Rhod** nanogels in PBS (◆), by comparison of **CS-Fluo-TPP/HA** (△) and **CS-TPP/HA-Rhod** (◯) in **CS-Fluo-TPP/HA-Rhod** nanogels in PBS (λ_ex_ = $\lambda_{ex}^{D}\;$ = 470 nm, CS-Fluo being the donor dye and HA-Rhod being the acceptor dye). (**b**) Fluorescence measurements of **CS-Fluo-TPP/HA-Rhod** nanogels ([A]/[D] ratio = 0.5) without (◆) and in the presence of hyaluronidase (×).

As shown, nanogels can be readily obtained by an ionotropic gelation process between functionalized hyaluronic acid solutions and chitosan ones, in the presence of tripolyphosphate (TPP) as a crosslinker [44]. We have previously demonstrated that these nanogels are very helpful to boost the performance of gadolinium chelates (GdCAs) used as contrast agents in MRI [31,32,34]. There remains a need for knowledge of the stability of these nanoassemblies and the synthesis of **HA-Rhod** polymers can be used to evaluate it by FRET. More precisely, FRET experiments have allowed to evaluate the molecular proximity of both polymers thanks to a fluorescent donor–acceptor pair. For this purpose, nanogels were synthesized by mixing **HA-Rhod** and **CS-Fluo** partners according to the conditions used for the synthesis of nanogels that encapsulate GdCAs. In these conditions, the ratio [A]/[D] was equal to 0.5. Since the degree of substitution of each polymer was low (DS_CS_ = 1%, DS_HA_ = 4.9%), the properties of each partner were not perturbed (i.e., the number of positive and negative charges carried by CS and HA, respectively) and the **CS-Fluo-TPP/HA-Rhod** nanogel formation was evidenced by DLS and ELS measurements (Z-ave = 115 nm, PDI = 0.21, ζ = 26 mV). The emission spectrum of **CS-Fluo-TPP/HA-Rhod** nanogel was then recorded after excitation at 470 nm, i.e., at the excitation wavelength of the donor dye, and compared to the ones of **CS-Fluo-TPP/HA** and **CS-TPP/HA-Rhod** nanogels (Figure 5a). The **CS-Fluo-TPP/HA-Rhod** nanogel fluorescence spectrum exhibited two signals at 525 and 591 nm attributed to fluorescein and rhodamine emissions respectively. By comparison to the **CS-TPP/HA-Rhod** nanogel luminescence spectrum recorded under similar conditions (after excitation at fluorescein wavelength at 470 nm), it was noticeable that the emission intensity of rhodamine signal at 591 nm in the **CS-Fluo-TPP/HA-Rhod** nanogel was greatly exalted. This was the fingerprint of an energy transfer between fluorescein and rhodamine and this FRET signal confirmed the close proximity of **CS-Fluo** and **HA-Rhod** within the **CS-Fluo-TPP/HA-Rhod** nanogel structure. This signal was persistent over a period of one month in PBS (a longer analysis period has not been tested), illustrating the stability of the nanogels under these conditions. Furthermore, FRET properties of **CS-Fluo-TPP/HA-Rhod** nanogels were tested in the presence of hyaluronidase enzyme (HA-ase). **CS-Fluo-TPP/HA-Rhod** nanogels were incubated at 37 °C in the presence of hyaluronidase HYAL-1 at a concentration of 60 ng·mL^−1^, which is the HYAL-1 concentration in human serum [46]. No changes in the FRET spectrum were detected (Figure 5b), which highlighted the stability of **CS-Fluo-TPP/HA-Rhod** nanogels under these physiological conditions.

## 3. Conclusions

To conclude, our objective in this work was to obtain functionalized HA with stealth, lipophilic, or fluorescent properties and to test their ability to form nanogels by ionic gelation with chitosan. Successful HA grafting was obtained in DMSO through a peptidic coupling between the amino-terminal group of the grafted synthons and the carboxylic moieties of protonated HA. In DMSO, the identification of the amidic function was most often straightforward and allowed the determination of the HA degree of substitution (DS_HA_). When this identification was not possible, a combination of ^1^H NMR and DOSY experiments was used. A series of functionalized HAs were then described and DS_HA_ seemed to cap to about 30–50% according to the grafted function except for rhodamine synthon for which DS_HA_ did not exceed 5%. This was probably due to the fact that the reactive rhodamine nitrogen atom was secondary, more sterically hindered, and then less reactive towards the peptidic coupling strategy.

Ionic gelation from all HA conjugates, whatever DS_HA_, proved to be efficient to provide CS-TPP/functionalized HA nanohydrogels having morphological characteristics compatible with biomedical applications. Ionic gelation was then used to synthesize nanohydrogels combining fluorescein-labeled chitosan and HA-Rhod. FRET experiments performed with the corresponding nanoassemblies that carried this fluorescent donor–acceptor pair allowed to demonstrate the close proximity of CS and HA polymers within the nanogel matrix. In the presence of physiological amounts of hyaluronidase, no modification of the FRET signal was observed which allowed to conclude a good stability of these nanohydrogels in a biological medium, which was a prerequisite to their use in biomedical applications.

## 4. Materials and Methods

### 4.1. Materials

Hyaluronic acid sodium salt (**HAs**, from *Streptococcus equi* M_W_ ~1.5–1.8 × 10^6^ Da), 1-[bis(dimethylamino)methylene]-1H-1,2,3-triazolo[4,5-b]pyridinium 3-oxid hexafluoro-phosphate (**HATU**) and hyaluronidase (HYAL-1 from bovine testes, 407 UI·mg^−1^) were purchased from Sigma-Aldrich (Saint Louis, MO, USA). Amino synthons involved in this work were *n*-octylamine **C8-NH_2_** (Alfa Aesar, Kandel, Germany), 1,1,1-trifluoropropylamine **TFP-NH_2_** (Sigma-Aldrich, Saint Louis, MO, USA), 2,5,8,11,14,17,20-heptaoxadocosan-22-amine **PEG_339_-NH_2_**, and methoxy-poly(ethylene)glycol-amine **PEG_2000_-NH_2_** (both synthons purchased from Iris Biotech GmbH, Marktredwitz, Germany). Amine-functionalized rhodamine **Rhod-NH** was prepared from Rhodamine B (purchased from Sigma Aldrich, Saint Louis, MO, USA) following a literature-inspired method [43]. For calculations, **HAs** repetitive unit molecular mass was considered to be M_W_ in average (**HAs**) = 401 g·mol^−1^. Amberlite™ IR 120 ion exchange resin was purchased from Fluka (Buchs, Switzerland). Vivaspin^®^ 20 ultrafiltration tubes (MWCO 10,000 Da) were purchased from Sartorius (Göttingen, Germany). Ultrafiltration experiments were realized with an Allegra X-30 Centrifuge (Beckman-Coulter) (7500 rpm, between 45 and 60 min, at room temperature). DMSO-*d*_6_ was purchased from Eurisotop. The syntheses of organic nanoparticles by the ionic gelation method were performed using chitosan (**CS**, Sigma-Aldrich, low viscosity, deacetylation degree 86% determined by ^1^H NMR spectroscopy [47,48,49]) and sodium tripolyphosphate (TPP, Acros Organics). Sterile water for injections (Laboratoire Aguettant, Lyon, France) was systematically used for nanoparticle preparations and analyses. All products were used as received, without further purification.

Polymers and copolymers (**HAp**, **HA-C8**, **HA-TFP**, **HA-PEG_339_**, **HA-PEG_2000_**, **HA-Rhod**) were characterized by means of FTIR (Thermo Scientific™ Nicolet™ iS5 spectrometer equipped with ATR iD5 accessory), ^1^H, and ^19^F NMR spectroscopies (Bruker Avance III (^1^H—500 MHz, ^19^F—470.6 MHz) spectrometer) at 318K with DMSO-*d*_6_ as the solvent. The diffusion coefficients of different materials (**HAp**, **HA-C8**, **HA-Rhod**, **C8-NH_2_**, **Rhod-NH**) were determined by DOSY experiments (diffusion ordered spectroscopy) on an Avance II 500 spectrometer (Bruker).

### 4.2. Syntheses and Purifications of Functionalized HA

HA functionalization occurs in two steps: first, the protonation of carboxylate groups to make it soluble in DMSO [50,51] and second, peptidic coupling with the amine, using HATU as the coupling agent.

#### 4.2.1. Conversion of Sodium Hyaluronate HAs into Its Protonated Form HAp

At neutral pH, HA is in the form of a sodium salt and is referred to as sodium hyaluronate (**HAs**). In order to transform it in its protonated form, ion exchange was undertaken similarly as described by Vasi et al. [20]. Sodium hyaluronate (1.00 g), dissolved in demineralized water (400 mL) was slowly eluted through an Amberlite™ IR 120 ion exchange resin (25 mL in dry volume, dispersed in 50 mL of demineralized water) conditioned under HCl form (by addition of 50 mL HCl 1M and then rinsing with 50 mL of demineralized water). The resulting solution was pre-concentrated under reduced pressure and freeze-dried to afford 0.92 g of protonated HA (yield 97%).

#### 4.2.2. General Method of HA Functionalization by Peptidic Coupling Reaction with Amine Synthons in DMSO

The starting compounds were separately dissolved in anhydrous DMSO: protonated HA (**HAp**, 80 mg, 0.21 mmol, 8 mL DMSO), **HATU** (40 mg, 0.105 mmol, 1 mL DMSO), and amine synthon (0.1 mmol, 1 mL DMSO). **HAp** solution was added in four glass vials (2 mL in each vial, 0.05 mmol), equipped with magnetic stirring bars, and previously purged with argon. Increasing quantities of **HATU** were then added in each vial (50, 100, 250, and 500 µL, corresponding to 0.1, 0.2, 0.5, and 1.0 equivalent, respectively) and followed by dilution with anhydrous DMSO (900, 800, 500, and 0 µL, respectively). HA was activated over 15 min and amine solution was added in each vial (50, 100, 250, and 500 µL, corresponding to 0.1, 0.2, 0.5, and 1.0 equivalent, respectively). After 24 h of reaction at room temperature, the solutions were transferred into 50 mL Falcon^®^ tubes and the addition of diethyl ether (27 mL into each tube) provoked the polymer precipitation. Functionalized HAs were then isolated by centrifugation and the corresponding solids were washed once more with diethyl ether (20 mL). After a second centrifugation, the products were dried under reduced pressure. Each product was then dissolved in 5 mL of 0.1 M HCl, transferred into a Vivaspin^®^ 20 tube (with MWCO 10,000 Da), diluted with 7 mL of demineralized water, and centrifuged at 6000 g. After two cycles of ultrafiltration, the final solutions were transferred into 15 mL Falcon^®^ tubes and freeze-dried. The sample was obtained by grafting 1 equiv. of octylamine on HA was not soluble in water. Three cycles of washing by centrifugation were applied in place of ultrafiltration.

### 4.3. Determination of Functionalized HA Degree of Substitution DS_HA_

The evaluation of the degree of substitution of HA (DS_HA_) on four categories of compounds (aliphatic, fluorinated, pegylated, and fluorescent amines (rhodamine)) was performed by ^1^H NMR methods. The functionalization with octylamine was chosen as a model reaction to develop the method for determining the DS_HA_ in the corresponding **HA-C8** polymers. Then, DS_HA_ was first determined by the integration of the newly formed amide proton ^1^H NMR signal, normalized to an acetamide NH signal of the HA backbone. This approach allowed to directly give a percentage of HA functionalized COOH groups. At this point, DOSY experiments were used to corroborate this percentage (*vide infra*). Then the integration approach was used to determine DS_HA_ in the case of **HA-TFP**, **HA-PEG_339_**, and **HA-PEG_2000_** polymers. Indeed, for these compounds, the peptidic coupling also generated a secondary amide bond, with a ^1^H signal that acts as a probe for the functionalization. In the case of **HA-Rhod** polymers, the newly formed amide bond is tertiary. For **HA-Rhod** polymers, DS_HA_ was calculated by comparison of the integrals of distinctive aliphatic protons peaks related to the introduced side groups and a HA acetamide methyl signal; these ratios were further corrected by DOSY analysis as described in one of our previous works [37].

For DOSY experiments, bipolar gradient pulses with two spoil gradients were used to measure the diffusion coefficients (BPP-LED pulse sequence). The value of the gradient pulse length δ was 2 or 4 ms depending on the samples, while the value of the diffusion time Δ was set to 150, 250, or 500 ms depending on the samples. The pulse gradients were incremented in 16 steps from 2% to 95% of the maximum gradient strength (53.5 G/cm) in a linear ramp and the temperature was set at 30 °C. Under these conditions, preliminary DOSY experiments were performed to determine **HA** and octylamine diffusion coefficients (D_HA_ and D_C8_, respectively). Values of 1.8 × 10^−11^ m^2^·s^−1^ and 6.0 × 10^−10^ m^2^·s^−1^, were obtained for HA and octylamine respectively.

Similar DOSY experiments were then performed with **HA-C8** polymers to characterize the diffusion coefficients of ungrafted and grafted C8 chains. The diffusion curves were extracted from **HA-C8** DOSY spectra for two peaks of C8 at 0.8 and 1.2 ppm and were characterized by two contributions: one coming from the ungrafted C8 (C8_UG_) which diffuses fast, and the other coming from the grafted C8 (C8_G_). Diffusion curves can thus be fitted with a bi-exponential equation taking into account the two contributions (Equation (1)) [52,53].

I = I_G_ exp[−γ^2^ g^2^ D_G_ δ^2^ (Δ − (δ/3) − (τ/2))] + I_UG_ exp[−γ^2^ g^2^ D_UG_ δ^2^ (Δ − (δ/3) − (τ/2))] (1)

where I_G_ and I_UG_ are the intensities at 0% gradient of grafted and not grafted C8, respectively, γ is the gyromagnetic ratio, g is the gradient strength, D_G_ and D_UG_ are the diffusion coefficients of grafted and ungrafted C8, respectively, δ is the gradient pulse length, Δ is the diffusion time, and τ is the interpulse spacing in the BPP-LED pulse sequence.

Assuming that the **HA-C8** molecular weight must be close to the one of HA (due first to the large difference between C8 and HA molecular weights), one can consider that C8_G_ (and then HA-C8) has the same diffusion coefficient as HA. During the fitting, D_G_ and D_UG_ were then fixed to values measured independently on HA and C8, respectively: D_HA_ = 1.8 × 10^−11^ m^2^·s^−1^, D_C8_ = 6.0 × 10^−10^ m^2^·s^−1^.

The values of I_G_ and I_UG_ extracted from the fitting allowed to calculate the percentage of the grafted C8 over the total amount of C8 (C8_G_/C8_T_):

(2) $$\frac{C8_{G}}{C8_{T}}=\frac{I_{G}}{I_{G}+I_{\mathrm{UG}}}\times 100$$

The percentage of C8 grafted to HA chains (DS_C8/HA_) was then calculated from ^1^H NMR and DOSY experiments:

(3) $${\mathrm{DS}}_{C8/\mathrm{HA}}=\;\%\frac{C8_{G}}{\mathrm{HA}}=\frac{I{\left(3H,\;C8\right)}_{0.87\;\mathrm{ppm}}}{I{\left(3H,\;\mathrm{HA}\right)}_{1.78\;\mathrm{ppm}}}\times \frac{I_{G}}{I_{G}+I_{\mathrm{UG}}\;}\times 100$$

where I represents the integration of the peaks indicated in brackets and I_G_ and I_UG_ stand for the intensities extracted from the DOSY experiments, for grafted and ungrafted C8, respectively.

The same procedure was applied to characterize **HA-Rhod** polymers and determine their substitution degrees DS. For that, the diffusion coefficient of rhodamine was measured and a value of 2.4 × 10^−10^ m^2^·s^−1^ was obtained, which corresponds to D_UG_ in Equation (1).

### 4.4. Preparation of Nanoparticles with Functionalized HA Polymers by Ionic Gelation and Characterizations

#### 4.4.1. CS-TPP/Functionalized HA Nanogel Synthesis

Functionalized HAs obtained in this study (**HA-PEG_339_**, **HA-PEG_2000_**, **HA-TFB**, **HA-Rhod**, 3.6 mg) were dissolved in water (3.15 mL) and allowed to stir overnight in the presence of NaOH (1.35 mL of NaOH 0.1 M) for deprotonation of the remaining carboxylic acid groups. Stock solutions of CS were prepared by dissolution of the CS powder (2.5 mg·mL^−1^) in a 10% (*m/v*) citric acid aqueous solution, or in a 10% (*v/v*) acetic acid solution, and stirred overnight. Insoluble residues were removed by centrifugation at 3800 rpm for 4 min at room temperature. CS-TPP/functionalized HA nanogels were obtained by an ionotropic gelation process. The polyanionic phase (4.5 mL), i.e., functionalized HA (0.8 mg·mL^−1^) and TPP (2.4 mg·mL^−1^) were added dropwise to the CS solution (9 mL) under sonication (750 W, amplitude 32%) to obtain nanosuspensions. At the end of the addition, magnetic stirring was maintained for 10 min. The removal of unreacted compounds was achieved by dialysis (Spectrapore^®^, MWCO 25 kDa, Spectrumlab) against water (3 × 12 h).

#### 4.4.2. Nanogels Characterization by Dynamic Light Scattering (DLS)

Averaged hydrodynamic diameters (Z-ave) of nanoparticles were determined by Dynamic Light Scattering (DLS) with a Zetasizer Nano ZS (Malvern Zetasizer Nano-ZS, Malvern Instruments, Worcestershire, UK). Polydispersity indexes (PdI) were determined by cumulant analysis. Each nanosuspension was analyzed in triplicate at 20 °C at a scattering angle of 173°, after 1/20 dilution in water. Water for injection was used as a reference dispersing medium. ζ-(zeta) potential data were collected through electrophoretic light scattering at 20 °C, 150 V, in triplicate for each sample, after 1/20 dilution in water. The instrument was calibrated with a Malvern—68 mV standard before each analysis cycle.

#### 4.4.3. Atomic Force Microscopy

CS-TPP/functionalized HA nanosuspensions were analyzed by Atomic Force Microscopy (AFM) in solution in order to afford minimum perturbation of the samples [54,55]. 35 µL of each nanosuspension was directly deposited on freshly cleaved mica disks. After 20 min of deposition at ambient temperature, the sample was rinsed several times in distilled water. Nanosuspensions were then imaged in distilled water, under manually operated PeakForce Tapping mode (PFT) on a Brüker Resolve setup (Billerica, MA, USA). The average PeakForce setpoint was set around 100 pN, which was found to be a good compromise to remain in good tracking conditions and to avoid particle damage. MSNL probes (Bruker, Billerica, MA, USA) with an average nominal spring constant of 0.07 N/m were used. For each type of nanogel, three different samples were prepared and at least three different areas were imaged per sample to ensure the reproducibility of the measurements. For image processing, all images were analyzed and particle diameters were estimated using Nanoscope Analysis 1.8 (Bruker, Billerica, MA, USA). For the particle analysis, only individual and well-distinguished nanoparticles were taken into consideration and to obtain reliable statistical results.

The AFM setup is directly coupled to a confocal Zeiss LSM 800 microscope (Oberkochen, Germany) allowing to correlate fluorescent and AFM images.

#### 4.4.4. Evaluation of CS-Fluo-TPP/HA-Rhod Nanogels Stability by Förster Resonance Energy Transfer (FRET) Experiments

**CS-Fluo-TPP/HA-Rhod** nanogels for which the [A]/[D] ratio was equal to 0.5 were synthesized according to the protocol previously described. **HA-Rhod** (as the acceptor dye 3.6 mg, DS_HA_ = 4.9%) was dissolved in water (3.15 mL) and allowed to stir overnight in the presence of NaOH (1.35 mL of NaOH 0.1 M) as previously described. Chitosan grafted with the fluorescein probe (**CS-Fluo**, as the donor dye, DS_CS_ = 1.0% [34]) was prepared by dissolution of the **CS-Fluo** powder (2.5 mg.mL^−1^) in a 10% (*m/v*) citric acid aqueous solution and stirred overnight. Insoluble residues were removed by centrifugation at 3800 rpm for 4 min at room temperature.

For FRET measurements, nanosuspensions were diluted 10-fold in ultrapure water, to be in the concentration range suitable for analysis. At this dilution, nanoparticles exhibited the same morphological characteristics as the raw suspensions, as confirmed by DLS measurements. Fluorescence measurements were conducted on an Edinburg FLS100 spectrophotometer. The fluorescence emission in response to an excitation at 470 nm was recorded between 480 and 800 nm (with Δλ_exc_ = 1.6 nm and Δλ_em_ = 1.8 nm), using in a 10 mm thick quartz cuvette (Hëllma) and ultrapure water as a reference. The FRET signal was detected at 600 nm. For experiments in the presence of the enzyme, HA-ase solution at 60 ng·mL^−1^ and nanogels suspensions were pre-heated at 37 °C for 10 min. **CS-Fluo-TPP/HA-Rhod** nanogels and HA-ase were then mixed and incubated at 37 °C for 1 h 30 min. Then, the nanogels in the presence of HA-ase were cooled at 4 °C and characterized by DLS and fluorescence measurements, as previously described.

## Figures and Tables

**Table 1:** Degrees of substitution of HA functionalized with *n*-octylamine.

	HA-C8a	HA-C8b	HA-C8c	HA-C8d
% mol (amine/COOH)_initial_	10%	20%	50%	100%
Association rate (AR) ^1^	4.0%	8.4%	23.4%	47.0%
Degree of substitution (DS_HA_) ^2^	1.9%	7.2%	22.4%	42.9%
Grafting degree (GD) ^3^	71%	92%	100%	100%
Degree of substitution (DS_HA_) ^4^	2.8%	7.7%	23.4%	47.0%

^1^ Found by integration of ^1^H NMR signal at 0.87 ppm normalized to acetamide CH_3_ peak at 1.78 ppm; ^2^ found by integration of ^1^H NMR signal at 7.9 ppm normalized to acetamide NH peaks at 7.4 ppm; ^3^ determined from DOSY experiments; ^4^ DS = AR × GD.

**Table 2:** Degrees of substitution of HA functionalized with oligo-ethyleneglycol-amines.

	HA-PEG_339_				HA-PEG_2000_			
Entries	a	b	c	d	a	b	c	d
% mol (amine/COOH)_initial_	10%	20%	50%	100%	10%	20%	50%	100%
Degree of substitution (DS_HA_) ^1^	2.4%	7.6%	15.7%	38.9%	1.3%	7.0%	14.1%	32.0%

^1^ Found by the integration of ^1^H NMR signal at 8.1 ppm normalized to acetamide NH peaks at 7.4 ppm.

**Table 3:** Degrees of substitution of HA functionalized with trifluoropropyl-amine.

	HA-TFPa	HA-TFPb	HA-TFPc	HA-TFPd
% mol (amine/COOH)_initial_	10%	20%	50%	100%
Degree of substitution (DS_HA_) ^a^	5.2%	6.5%	15.6%	29.2%

^a^ Found by integration of ^1^H NMR signal at 8.25 ppm normalized to acetamide NH peak at 7.4 ppm.

**Table 4:** Degrees of substitution of HA functionalized with rhodamine.

	HA-Rhod a	HA-Rhod b	HA-Rhod c	HA-Rhod d
% mol (amine/COOH)_initial_	10%	20%	50%	100%
Association rate (AR) ^1^	1.1%	1.9%	4.0%	7.6%
Grafting degree (GD) ^2^	25%	62%	71%	64%
Degree of substitution (DS_HA_) ^3^	0.3%	1.2%	2.8%	4.9%

^1^ Found by integration of ^1^H NMR signal at 1.2 ppm normalized to acetamide CH_3_ peak at 1.78 ppm; ^2^ determined from DOSY experiments; ^3^ DS = AR × GD.

**Table 5:** Intensity weighted (Z-average) diameters, polydispersity indexes (PdI), and zeta potential (ζ) of CS-TPP/functionalized HA nanoparticles according to HA degree of substitution (DS_HA_).

Synthon	DS_HA_ [%]	Z-Average ± sd (nm)	PdI ± sd	ζ ± sd (mV)
**PEG_339_**	2.4	132 ± 2	0.19 ± 0.01	+26 ± 3
	7.6	128 ± 1	0.18 ± 0.02	+23 ± 4
	15.7	138 ± 2	0.19 ± 0.01	+24 ± 3
	38.9	128 ± 1	0.17 ± 0.01	+29 ± 3
**PEG_2000_**	1.3	141 ± 2	0.18 ± 0.02	+26 ± 4
	7	149 ± 1	0.18 ± 0.01	+21 ± 3
	14.1	137 ± 2	0.19 ± 0.02	+24 ± 4
	32	146 ± 1	0.18 ± 0.01	+24 ± 3
**TFB**	5.2	153 ± 3	0.18 ± 0.02	+28 ± 3
	6.5	140 ± 2	0.20 ± 0.01	+25 ± 4
	15.6	148 ± 3	0.18 ± 0.02	+23 ± 3
	29.2	146 ± 3	0.20 ± 0.01	+23 ± 3
**Rhod**	0.3	137 ± 3	0.18 ± 0.01	+22 ± 3
	1.2	147 ± 2	0.18 ± 0.02	+23 ± 4
	2.8	148 ± 3	0.20 ± 0.01	+23 ± 3
	4.9	141 ± 3	0.18 ± 0.02	+21 ± 4
No synthon	0	139 ± 2	0.18 ± 0.01	+22 ± 3

## Data Availability

Not applicable.

## Supplementary Materials

The following files are available free of charge. The following supporting information can be downloaded at: https://www.mdpi.com/article/10.3390/gels8030182/s1, Figure S1. Diffusion curves extracted from the DOSY experiments recorded on **HA-C8** samples for the peaks of octylamine at 0.8 and 1.2 ppm. Diffusion curves extracted from DOSY experiments run on HA and C8 separately were added for comparison. Figure S2. Evolution of DS_HA_ according to increasing C8-NH_2_/COOH_HA_ initial ratios. Figure S3. FTIR spectra of PEG_339_ functionalized HA samples: (a) carbonyl stretching vibration region (1480–1820 cm^−1^) and b) C-H stretching vibration region (2700–3000 cm^−1^). Figure S4. FTIR spectra of PEG_2000_ functionalized HA samples: (a) carbonyl stretching vibration region (1480–1820 cm^−1^) and (b) C-H stretching vibration region (2700–3000 cm^−1^). Figure S5. Structure and ^1^H NMR spectra of PEG_339_ functionalized HA: **HAp**, **HA-PEG_339_a**, **HA-PEG_339_b**, **HA-PEG_339_c**, and **HA-PEG_339_d**, for initial molar ratios (amine/COOH) = 0 (bottom), 10, 20, 50, and 100 (top) % respectively (500 MHz, 318 K, DMSO-*d_6_*). Figure S6. Structure and ^1^H NMR spectra of PEG_2000_ functionalized HA: **HAp**, **HA-PEG_2000_a**, **HA-PEG_2000_b**, **HA-PEG_2000_c**, and **HA-PEG_2000_d**, for initial molar ratios (amine/COOH) = 0 (bottom), 10, 20, 50, and 100 (top) % respectively (500 MHz, 318 K, DMSO-*d_6_*). Figure S7. Evolution of DS_HA_ according to increasing PEG_339_-NH_2_/COOH_HA_ initial ratios. Figure S8. Evolution of DS_HA_ according to increasing PEG_2000_-NH_2_/COOH_HA_ initial ratios. Figure S9. FTIR spectra of TFP functionalized HA samples: (a) carbonyl stretching vibration region (1480–1820 cm^−1^) and (b) C-H stretching vibration region (2700–3000 cm^−1^). Figure S10. Structure and ^1^H NMR spectra of trifluoropropyl functionalized HA: **HAp**, **HA-TFPa**, **HA-TFPb**, **HA-TFPc**, and **HA-TFPd**, for initial molar ratios (amine/COOH) = 0 (bottom), 10, 20, 50, and 100 (top) % respectively (500 MHz, 318 K, DMSO-*d_6_*). Figure S11. 1D diffusion-filtered ^19^F NMR spectra of **HA-TFPa**, **HA-TFPb**, **HA-TFPc, HA-TFPd**, and **TFP-NH_2_** with a gradient *g* of 2% and 95%. Figure S12. Evolution of DS_HA_ according to increasing C8-TFP/COOH_HA_ initial ratios. Figure S13. FTIR spectra of rhodamine functionalized HA samples: (a) carbonyl stretching vibration region (1480–1820 cm^−1^) and (b) C-H stretching vibration region (2700–3000 cm^−1^). Figure S14. Structure and ^1^H NMR spectra of rhodamine B functionalized HA (500 MHz, 318 K, NS = 32); from bottom to up: **HAp**, **HA-Rhoda**, **HA-Rhodb**, **HA-Rhodc**, and **HA-Rhodd**. Figure S15. Evolution of DS_HA_ according to increasing Rhod-NH/COOH_HA_ initial ratios. Figure S16: topographical AFM images of (a) CS-TPP/HA-PEG_2000_, (b) CS-TPP/HA-Rhod, and (c) CS-TPP/HA (control) NGs. Figure S17: Coupled AFM and Confocal images of CS-TPP/HA-Rhod nanogels.
