# Supplementary material for: Synthesis and Characterization of Conjugated Hyaluronic Acids. Application to Stability Studies of Chitosan-Hyaluronic Acid Nanogels Based on Fluorescence Resonance Energy Transfer
Source: Gels. 2022 Mar 15;8(3):182. doi: 10.3390/gels8030182 (PMC8949952; doi:10.3390/gels8030182)
Supplement: Supplementary file 1 [file gels-08-00182-s001.zip › gels-1627800-Supplementary.pdf]

gels

Supplementary Materials

Synthesis and Characterization of Conjugated Hyaluronic Acids. Application to Stability Studies of Chitosan-Hyaluronic Acid Nanogels Based on Fluorescence Resonance Energy Transfer

Volodymyr Malystkyi *, Juliette Moreau, Maité Callewaert, Céline Henoumont, Cyril Cadiou, Cécile Feuillie, Sophie Laurent, Michael Molinari and Françoise Chuburu *

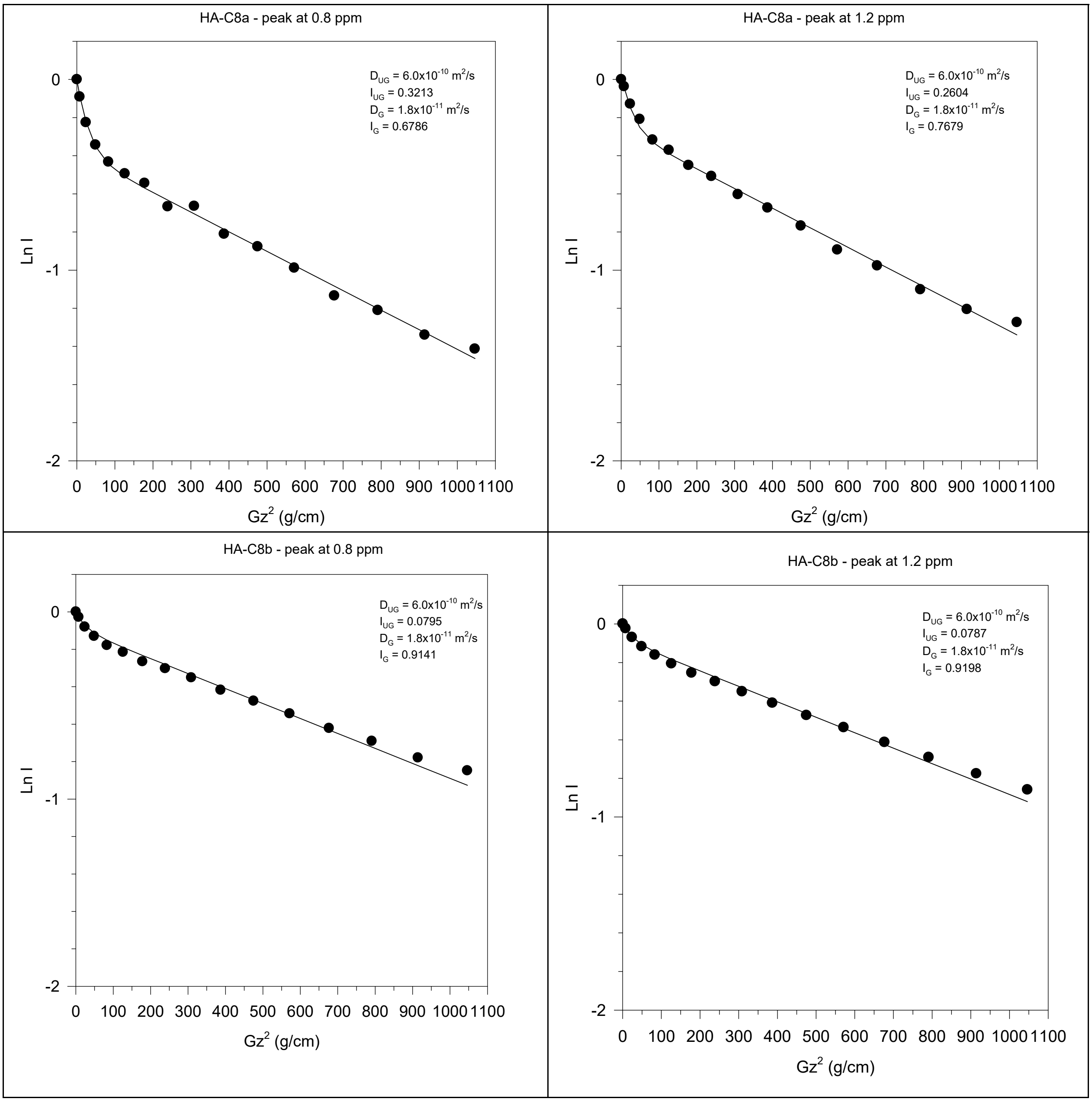

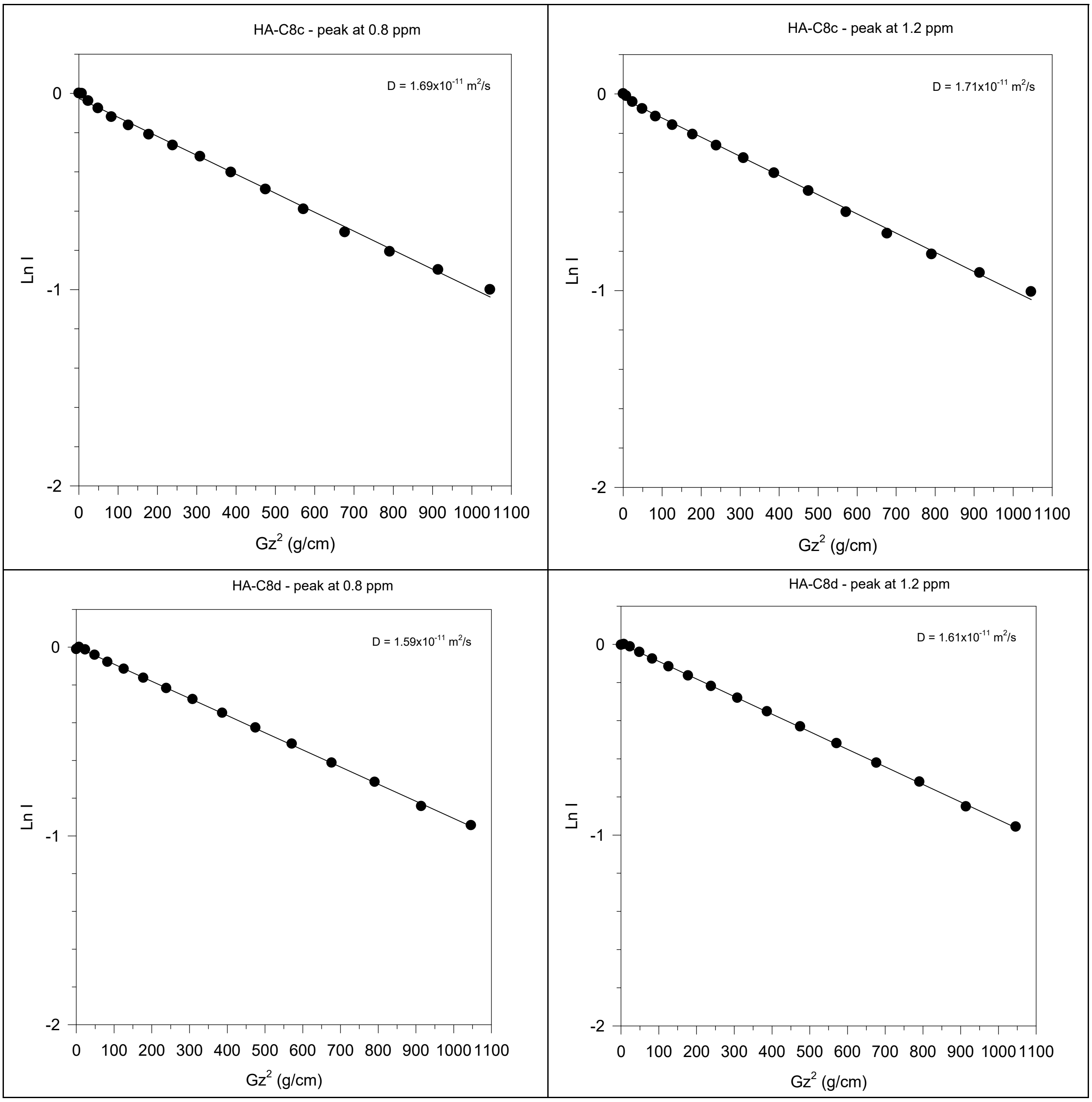
HA-C8c - peak at 0.8 ppm
D = 1.69x10-11 m2/s
HA-C8c - peak at 1.2 ppm
D = 1.71x10-11 m2/s
HA-C8d - peak at 0.8 ppm
D = 1.59x10-11 m2/s
HA-C8d - peak at 1.2 ppm
D = 1.61x10-11 m2/s
Ln I
Gz2 (g/cm)
0
-1
-2
0 100 200 300 400 500 600 700 800 900 1000 1100

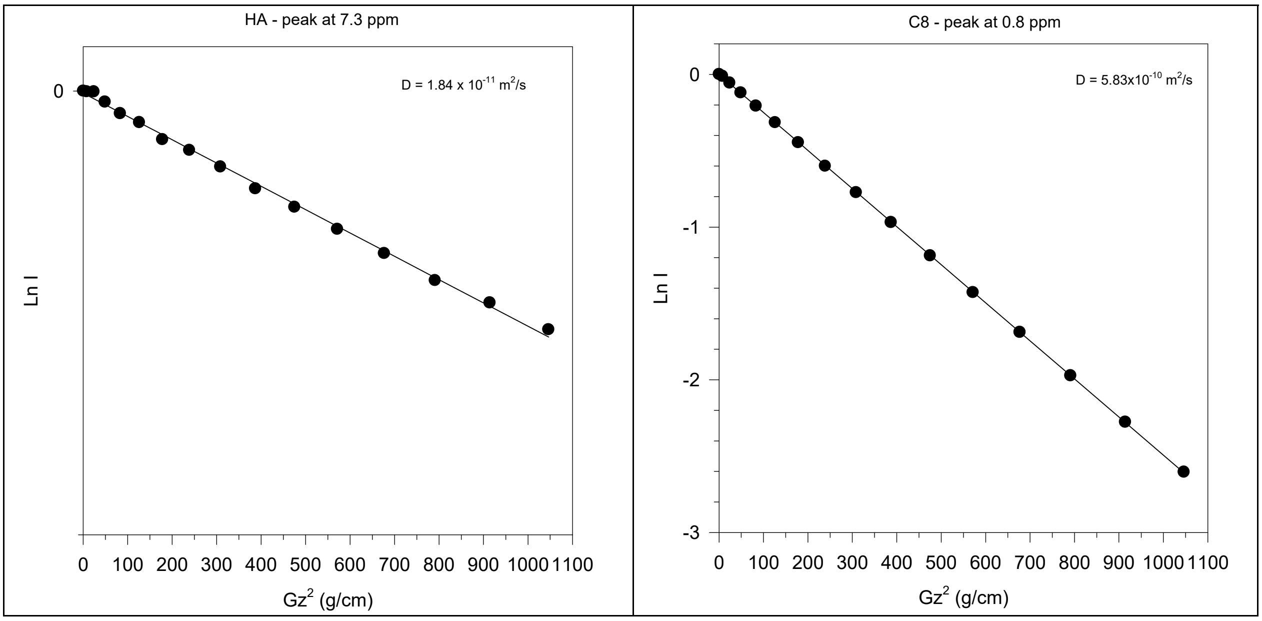

Figure S1. Diffusion curves extracted from the DOSY experiments recorded on **HA-C8** samples for the peaks of octylamine at 0.8 and 1.2 ppm. Diffusion curves extracted from DOSY experiments run on HA and C8 separately were added for comparison.

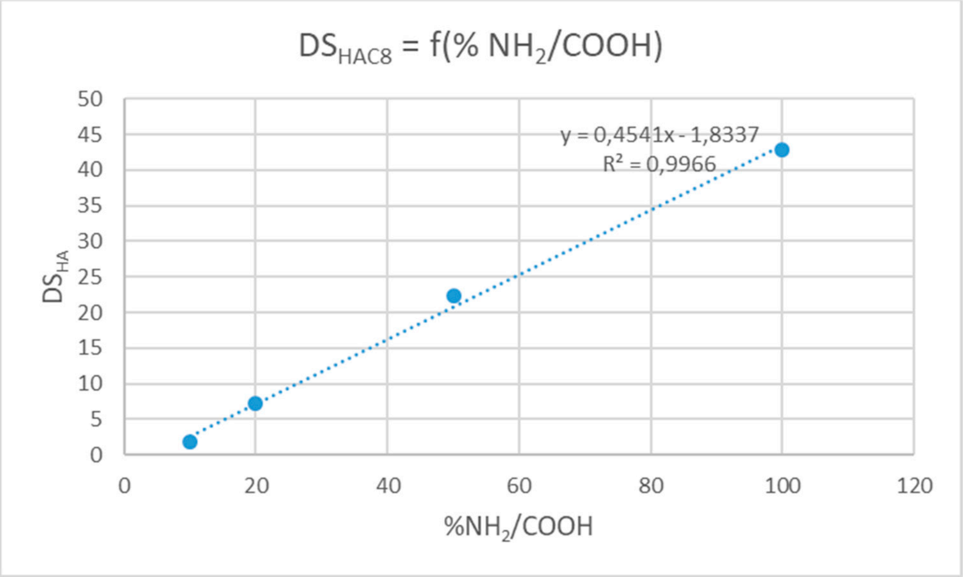

Figure S2. Evolution of DS_{HA} according to increasing C8-NH_2/$COOH_{HA}$ initial ratios.

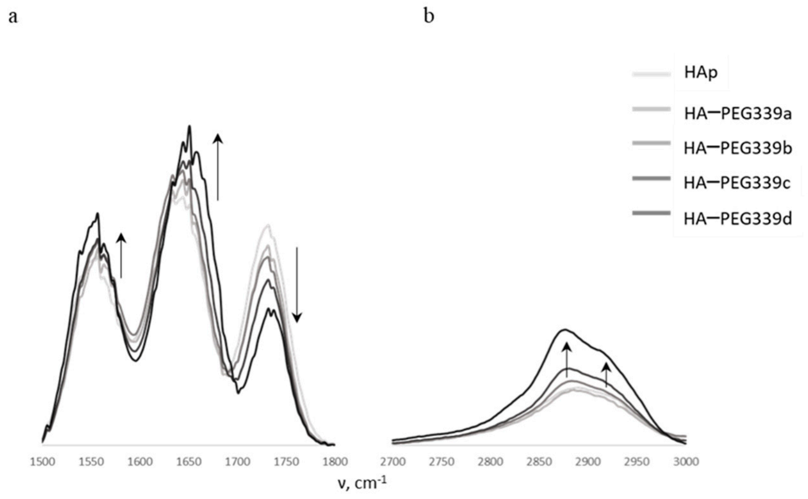

Figure S3. FTIR spectra of PEG_{339} functionalized HA samples: (**a**) carbonyl stretching vibration region (1480–1820 cm^{-1}) and (**b**) C–H stretching vibration region (2700–3000 cm^{-1}).

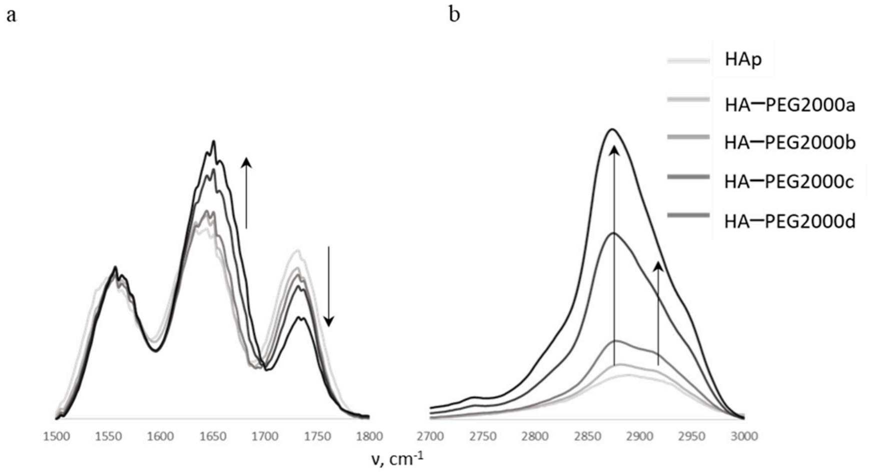

Figure S4. FTIR spectra of PEG_{2000} functionalized HA samples: (**a**) carbonyl stretching vibration region (1480–1820 cm^{-1}) and (**b**) C–H stretching vibration region (2700–3000 cm^{-1}).

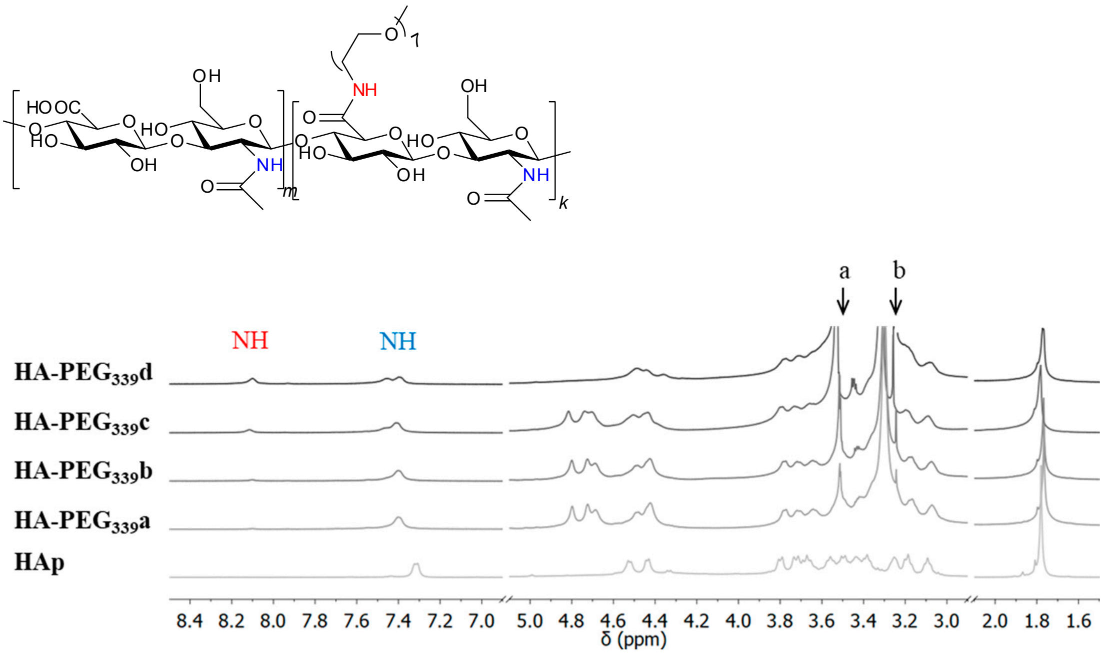

Figure S5. Structure and ^{1}H NMR spectra of PEG_{339} functionalized HA: **HAp**, **HA-PEG$_{339}$a**, **HA-PEG$_{339}$b**, **HA-PEG$_{339}$c**, and **HA-PEG$_{339}$d**, for initial molar ratios (amine/COOH) = 0 (bottom), 10, 20, 50, and 100 (top) % respectively (500 MHz, 318 K, DMSO-d_6). The signals corresponding to oligomeric ethylene glycol units and terminal methoxy groups were clearly observed at 3.51 ppm (signal **a**) and 3.24 ppm (signal **b**) respectively

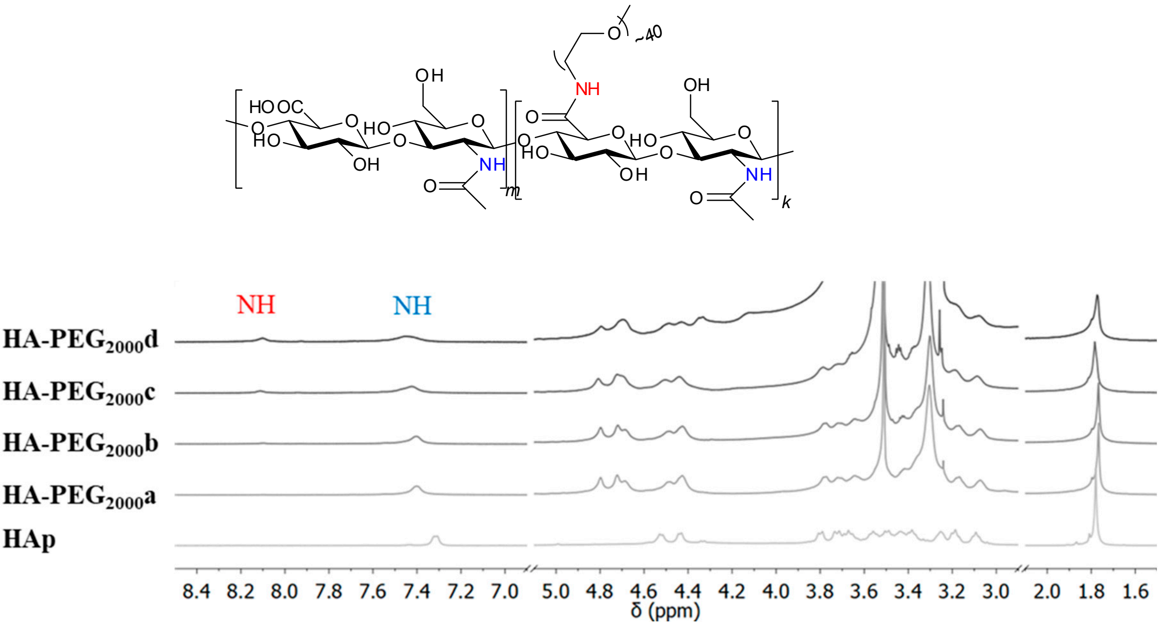

Figure S6. Structure and ^{1}H NMR spectra of PEG_{2000} functionalized HA: **HAp**, **HA-PEG$_{2000}$a**, **HA-PEG$_{2000}$b**, **HA-PEG$_{2000}$c**, and **HA-PEG$_{2000}$d**, for initial molar ratios (amine/COOH) = 0 (bottom), 10, 20, 50, and 100 (top) % respectively (500 MHz, 318 K, DMSO-d_6).

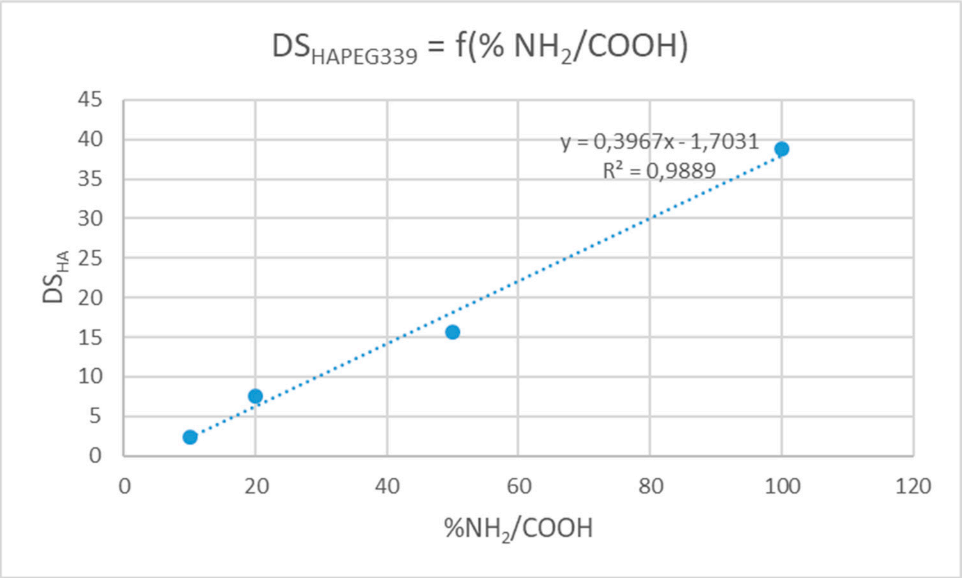

Figure S7. Evolution of DS_{HA} according to increasing PEG_{339}-NH_2/$COOH_{HA}$ initial ratios.

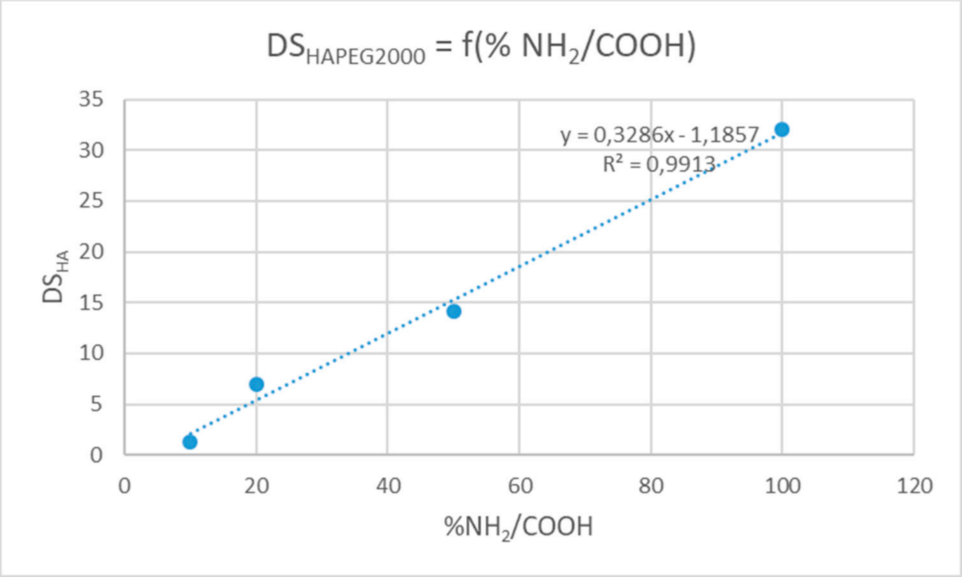

Figure S8. Evolution of DS_{HA} according to increasing PEG_{2000}-NH_2/$COOH_{HA}$ initial ratios.

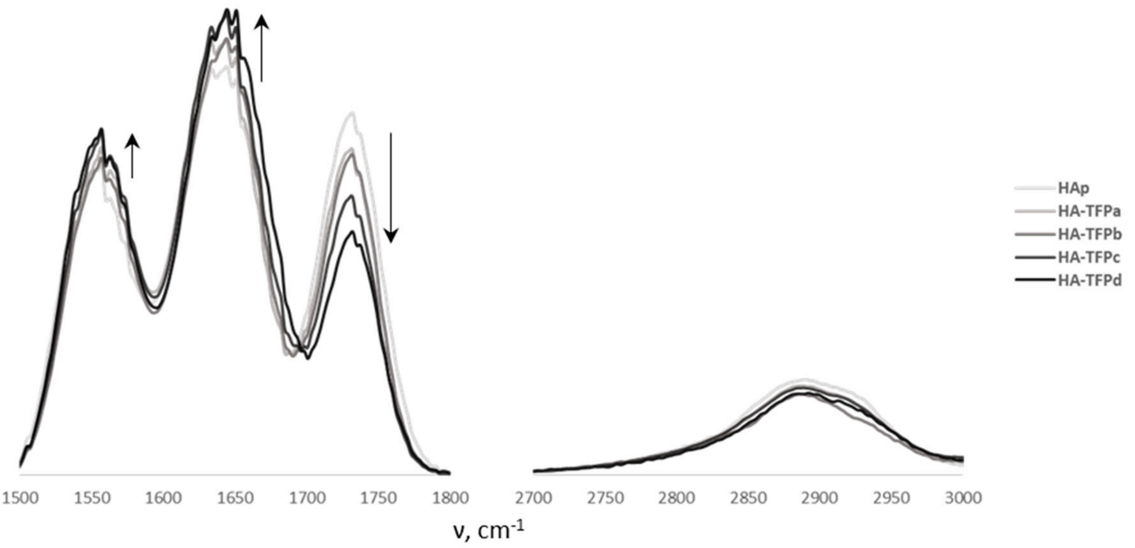

(a) (b)

Figure S9. FTIR spectra of TFP functionalized HA samples: (**a**) carbonyl stretching vibration region (1480–1820 cm^{-1}) and (**b**) C–H stretching vibration region (2700–3000 cm^{-1}).

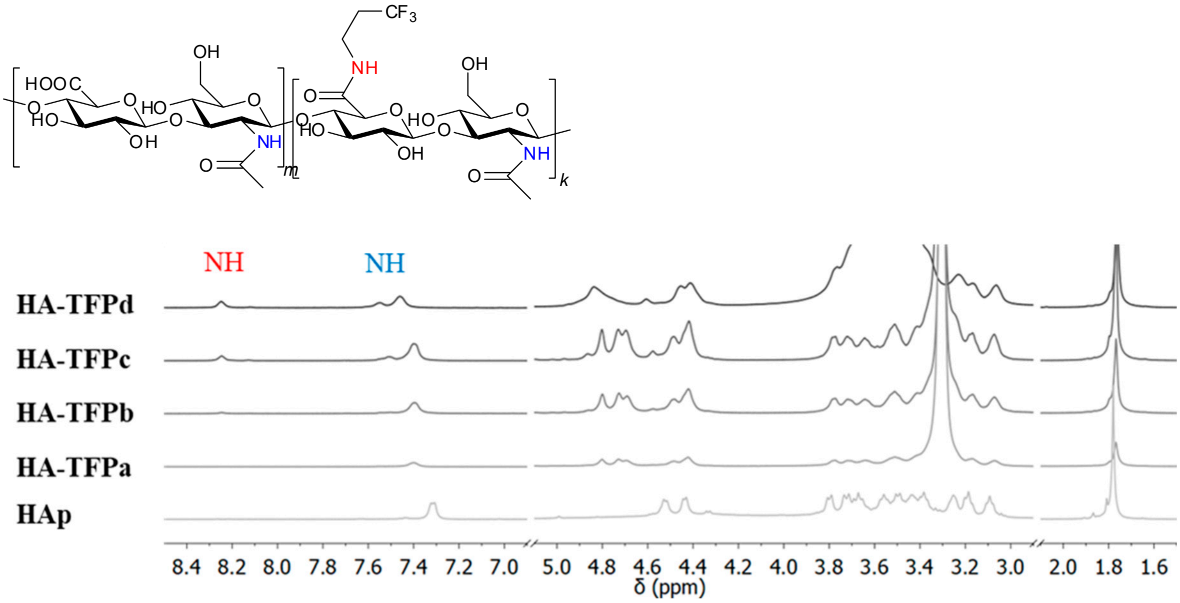

Figure S10. Structure and ^{1}H NMR spectra of trifluoropropyl functionalized HA: **HAp**, **HA-TFPa**, **HA-TFPb**, **HA-TFPc**, and **HA-TFPd**, for initial molar ratios (amine/COOH) = 0 (bottom), 10, 20, 50, and 100 (top) % respectively (500 MHz, 318 K, DMSO-d_6).

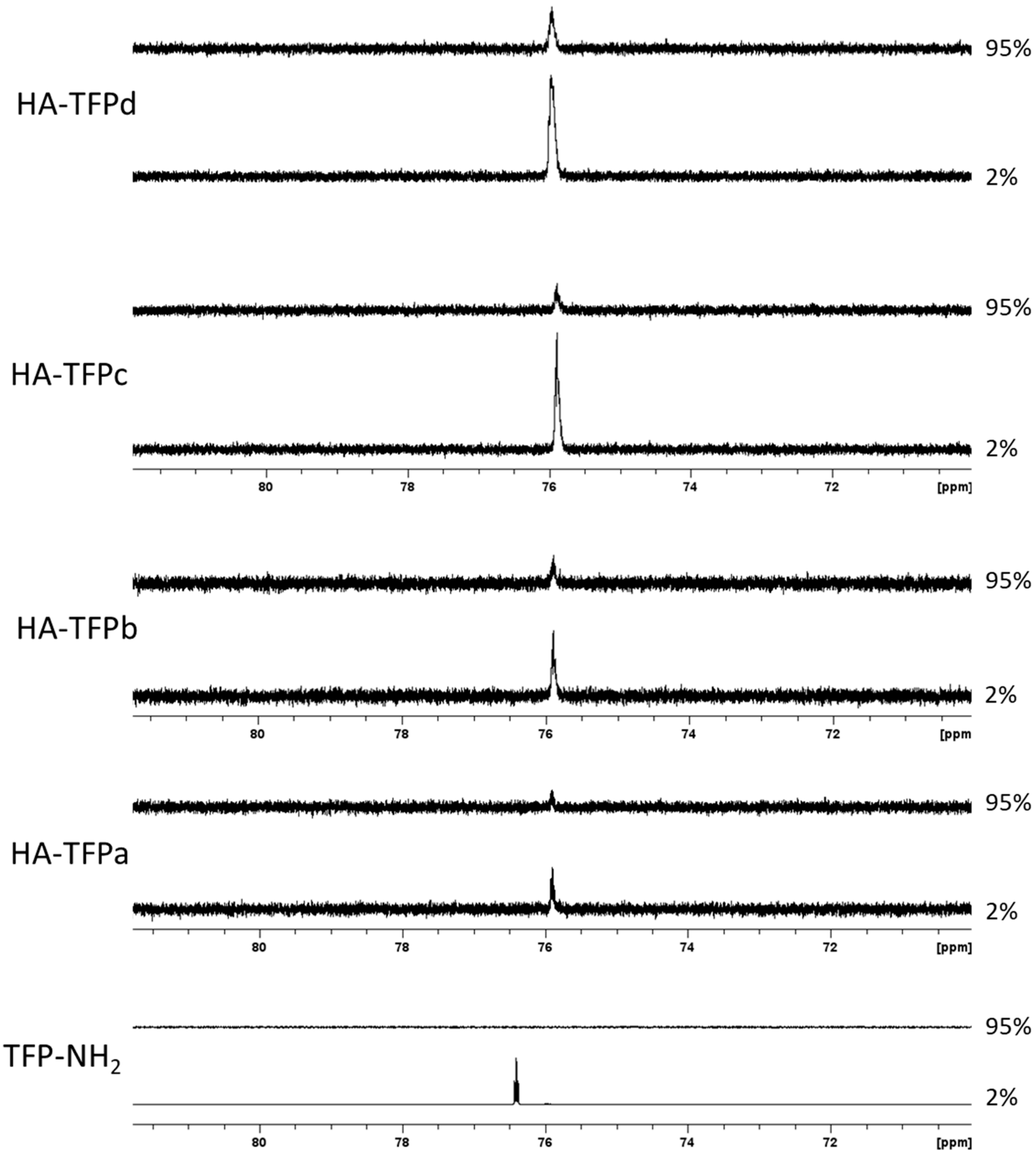

Figure S11. 1D diffusion-filtered ^{19}F NMR spectra of **HA-TFPa**, **HA-TFPb**, **HA-TFPc, HA-TFPd**, and **TFP-NH2** with a gradient *g* of 2% and 95%.

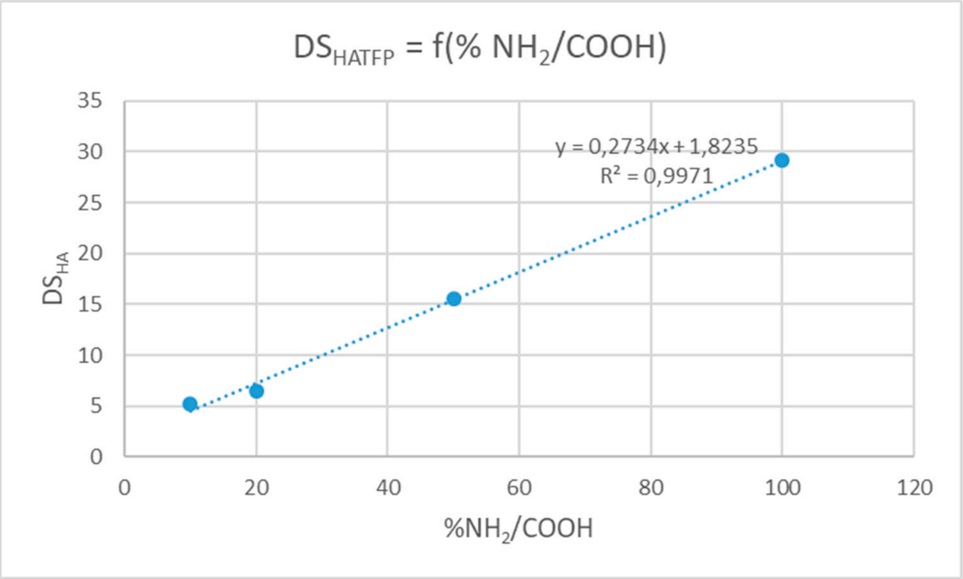

Figure S12. Evolution of DS_{HA} according to increasing TFP–NH_2/$COOH_{HA}$ initial ratios.

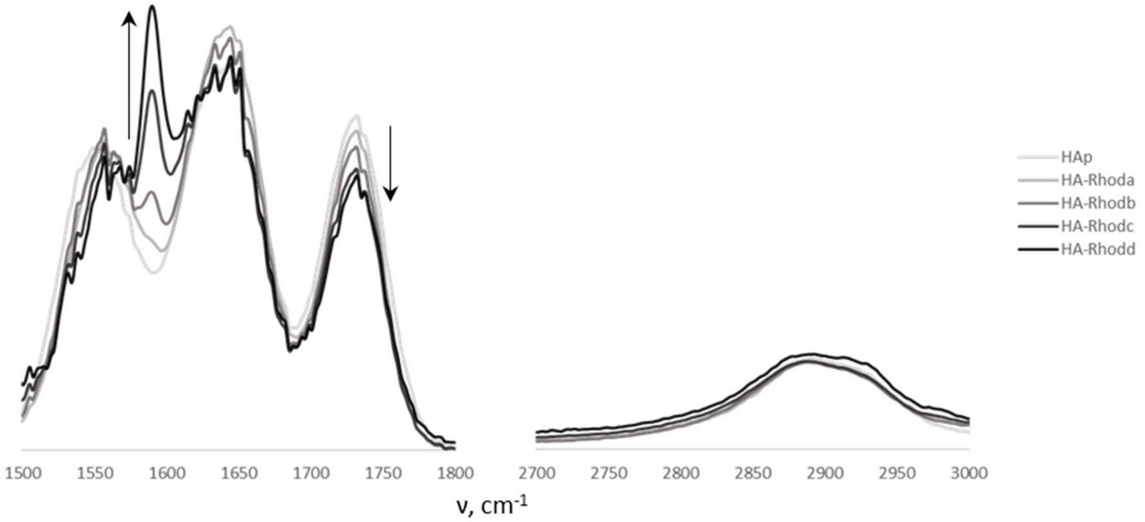

Figure S13. FTIR spectra of rhodamine functionalized HA samples: a) carbonyl stretching vibration region (1480–1820 cm^{-1}) and b) C–H stretching vibration region (2700–3000 cm^{-1}).

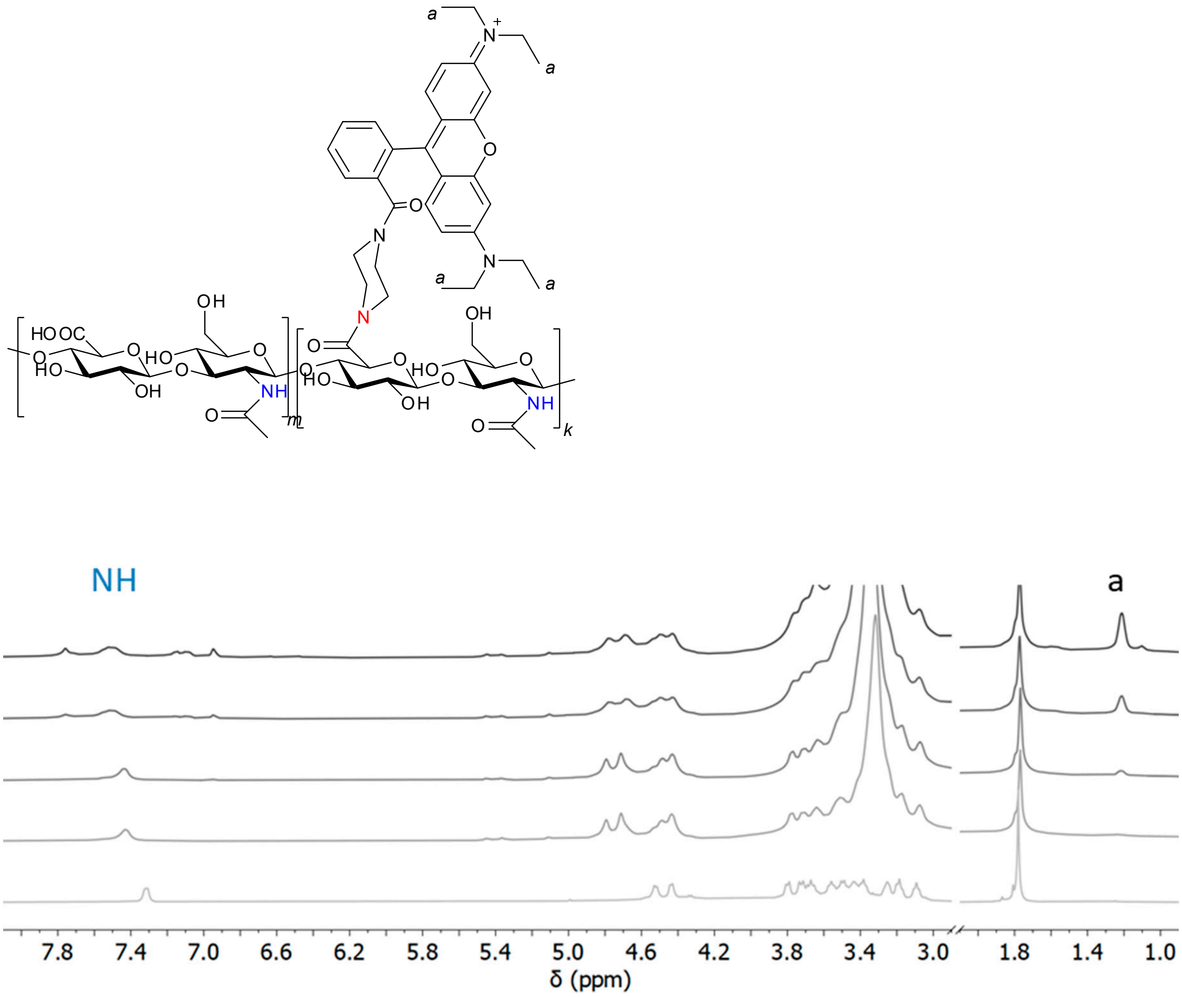

Figure S14. Structure and ^{1}H NMR spectra of rhodamine B functionalized HA (500 MHz, 318 K, NS = 32); from bottom to up: **HAp**, **HA-Rhoda**, **HA-Rhodb**, **HA-Rhodc**, and **HA-Rhodd**. Signal a at the high field corresponds to 12 H from two diethylamino groups of rhodamine.

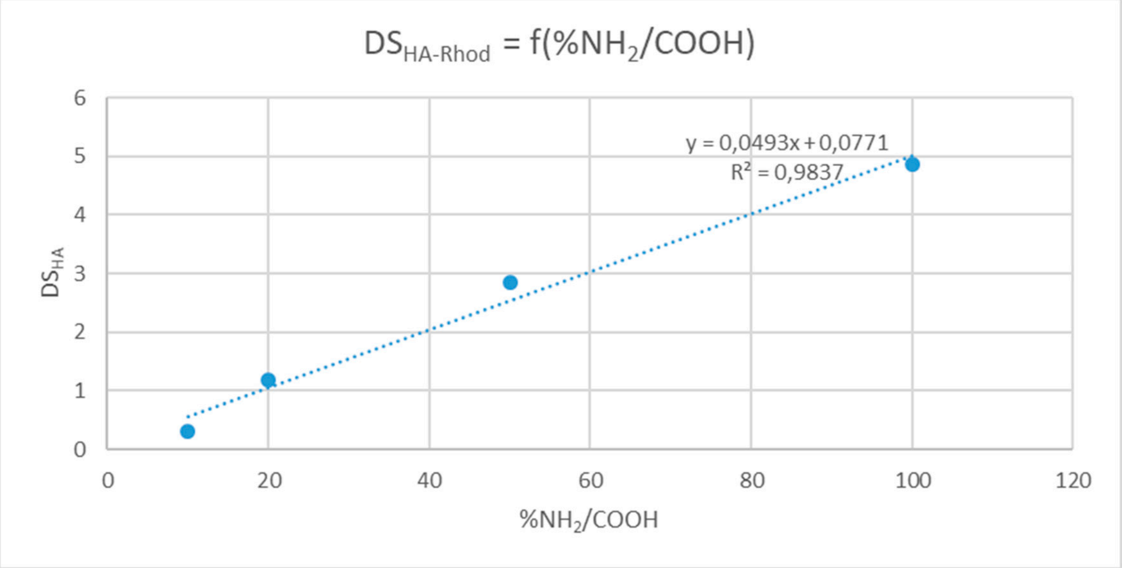

Figure S15. Evolution of DS$_{HA}$ according to increasing Rhod-NH/COOH$_{HA}$ initial ratios.

a

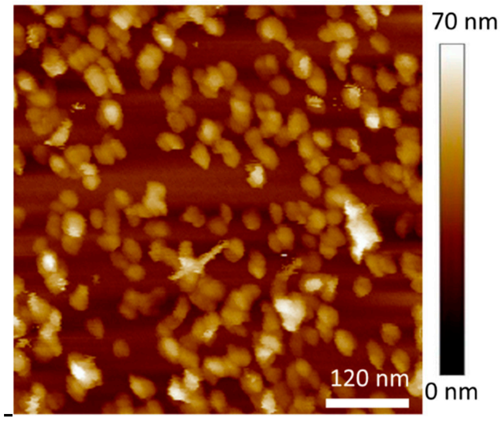

b-

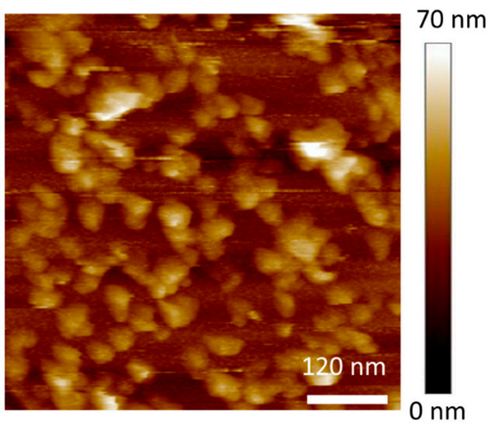

$30 < \phi_{AFM} < 40$ nm

$40 < \phi_{AFM} < 60$ nm

c-

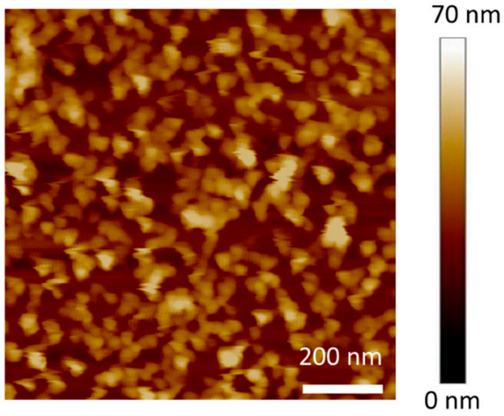

$40 < \phi_{AFM} < 70$ nm

Figure S16. topographical AFM images of (**a**) CS-TPP/HA-PEG$_{2000}$, (**b**) CS-TPP/HA-Rhod, and (**c**) CS-TPP/HA (control) NGs.

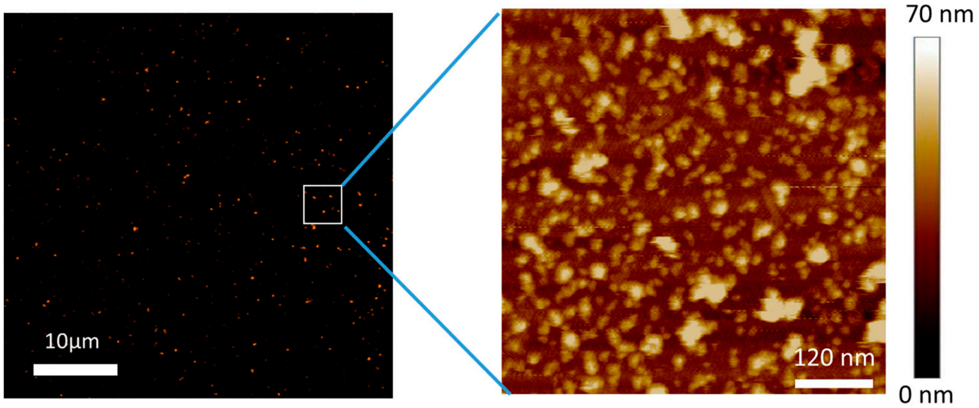

Figure S17. Coupled AFM and Confocal images of CS-TPP/HA-Rhod nanogels.
